# Modeling subjective relevance in schizophrenia and its relation to aberrant salience

**DOI:** 10.1371/journal.pcbi.1006319

**Published:** 2018-08-10

**Authors:** Teresa Katthagen, Christoph Mathys, Lorenz Deserno, Henrik Walter, Norbert Kathmann, Andreas Heinz, Florian Schlagenhauf

**Affiliations:** 1 Charité - Universitätsmedizin Berlin, corporate member of Freie Universität Berlin, Humboldt-Universität zu Berlin, and Berlin Institute of Health, Department of Psychiatry and Psychotherapy CCM, Berlin, Germany; 2 Berlin School of Mind and Brain, Humboldt-Universität zu Berlin, Berlin, Germany; 3 Scuola Internazionale Superiore di Studi Avanzati (SISSA), Trieste, Italy; 4 Max Planck UCL Centre for Computational Psychiatry and Ageing Research, London, United Kingdom; 5 Wellcome Trust Centre for Neuroimaging at UCL, London, United Kingdom; 6 Department of Child and Adolescent Psychiatry, Psychotherapy and Psychosomatics, University of Leipzig, Leipzig, Germany; 7 Max-Planck-Institute for Human Cognitive and Brain Sciences, Leipzig, Germany; 8 Department of Psychology, Humboldt-Universität zu Berlin, Berlin, Germany; Brain and Spine Institute (ICM), FRANCE

## Abstract

In schizophrenia, increased aberrant salience to irrelevant events and reduced learning of relevant information may relate to an underlying deficit in relevance detection. So far, subjective estimates of relevance have not been probed in schizophrenia patients. The mechanisms underlying belief formation about relevance and their translation into decisions are unclear. Using novel computational methods, we investigated relevance detection during implicit learning in 42 schizophrenia patients and 42 healthy individuals. Participants underwent functional magnetic resonance imaging while detecting the outcomes in a learning task. These were preceded by cues differing in color and shape, which were either relevant or irrelevant for outcome prediction. We provided a novel definition of relevance based on Bayesian precision and modeled reaction times as a function of relevance weighted unsigned prediction errors (UPE). For aberrant salience, we assessed responses to subjectively irrelevant cue manifestations. Participants learned the contingencies and slowed down their responses following unexpected events. Model selection revealed that individuals inferred the relevance of cue features and used it for behavioral adaption to the relevant cue feature. Relevance weighted UPEs correlated with dorsal anterior cingulate cortex activation and hippocampus deactivation. In patients, the aberrant salience bias to subjectively task-irrelevant information was increased and correlated with decreased striatal UPE activation and increased negative symptoms. This study shows that relevance estimates based on Bayesian precision can be inferred from observed behavior. This underscores the importance of relevance detection as an underlying mechanism for behavioral adaptation in complex environments and enhances the understanding of aberrant salience in schizophrenia.

## Author summary

Schizophrenia patients display deficits in the appropriate attribution of meaningfulness to stimuli; such as aberrantly increased processing of irrelevant and insufficient processing of relevant information. We aimed to investigate the subjective nature of relevance detection and its deficit in schizophrenia and developed an implicit learning paradigm that allowed for parallel learning from relevant and irrelevant information. Based on the idea that subjective relevance might be captured by Bayesian precision we set up different computational models of how subjective relevance guides learning and behavioral adaptation. We found that subjects use Bayesian precision to estimate stimulus relevance in order to integrate multidimensional information and adapt more to the subjectively relevant stimuli. This relevance weighted adaptation correlated with brain activation within the salience network. Further, schizophrenia patients displayed an increased aberrant tendency to irrelevant events which related to decreased striatal coding of the relevant learning signal. To conclude, our findings demonstrate how individual beliefs about relevance can be inferred from computational models. Furthermore, we suggest that aberrant salience observed in patients with schizophrenia reflects an idiosyncratic bias in states of high subjective uncertainty.

## Introduction

Reduced differentiation between relevance and irrelevance, a disruption of salience attribution, is the key component of the aberrant salience hypothesis of psychosis [1–3]. According to this theoretical framework, neurobiological noise in terms of increased striatal dopamine turnover may cause the subjective experience of salience or meaningfulness in the absence of relevant contextual events that usually cause dopaminergic saliency signaling. This experience of aberrant salience is then attributed to random, irrelevant events that coincide with it and, thus, these irrelevant events turn subjectively meaningful. At the same time, chaotic aberrant salience signaling was proposed to blur the signal-to-noise ratio leading to decreased processing of contextually relevant events and the formation and maintenance of negative symptoms [4, 5]. This salience framework clearly renders the objective experimental measurement of (aberrant) salience challenging and highlights the subjective nature of relevance and salience attribution. This subjectivity aspect can be captured by modeling latent learning processes of individuals, which is a common approach for describing the processing of relevant information, for example, during reinforcement learning [6, 7]. However, while computational modeling has already revealed decreased learning from task relevant events in schizophrenia patients [4], this approach has not been applied to learning from relevant compared to irrelevant stimuli, which might shed further light on aberrant salience attribution.

Both constructs, relevance and salience, are closely intertwined. In general, cues can be considered salient based on their physical characteristics, or cues are subjectively salient when they have been learned to be relevant in a certain context. For instance, neutral cues that are learned to predict reward can turn subjectively salient. Here, we define salient cues as those that have been learned to reliably predict important outcomes. These associations between cues and outcomes are learned via prediction error (PE) signals that code the surprise and unexpectedness of events; or computationally, the difference between observation and prediction. Thus, an unexpected event elicits a large unsigned prediction error (PE; a directed PE would carry information about the valence/reward) and the corresponding event would turn salient to the individual. Prediction errors are used to update the predictive value (belief) of the preceding stimulus. On the neural level, unexpectedness correlates with brain responses in the so-called salience network [8–11]; such as the (dorsal) anterior cingulate cortex and the insula [8, 12–16], while some studies also reported unsigned PE signaling in the striatum [for a review see 17].

In multidimensional environments, multiple cues are potentially important and individuals have to adapt to relevant cues that have proven to be reliable or precise predictors. A cue is precise when it announces a specific event with a high probability. Correspondingly, irrelevant cues that are experienced to be noisy and unreliable (= uncertain) should be dismissed. This was investigated by recent learning studies using multisensory cues [18–20] where subjects were either instructed to find out the relevant cue feature or were told which information they had to focus on in order to achieve the task of choosing the correct stimulus for reward maximization. While applying computational modeling, these studies provided behavioral and neural evidence for learning from multiple sources of information by integrating these according to their respective subjective relevance. According to the principles of Bayesian learning [21–23], as incorporated in the Hierarchical Gaussian filter [HGF; 24], Bayesian precision reflects the computational mechanism capturing the reliability of a stimulus. We use precision as our definition of subjective (in the sense of *learned*) relevance in multidimensional environments. Furthermore, we probe the influence of this subjective relevance on prediction errors, i.e. when the subject knows that the environment is irrelevant he/she should no longer experience a large prediction error as salient. In line with this approach, a theoretical account proposed that aberrant precision coding underlies psychosis formation [5, 25–27].

In schizophrenia and presumably due to chaotic dopaminergic signaling, the detection of task relevant cues seems to be disturbed while irrelevant cues not carrying reliable information can gain high subjective salience [1, 2, 28]. This latter phenomenon of aberrant salience describes the subjective experience of patients characterized by random stimuli suddenly standing out and turning meaningful. According to the hypothesis, patients make sense of this aberrant salience experience by forming cognitive schemes that on the long run turn into delusional beliefs. However, though the aberrant salience concept offers high descriptive value and plausibility regarding clinical symptoms the behavioral quantification in experimental settings still remains challenging. So far, conclusions about increased aberrant salience attribution has been drawn from two kinds of findings both related to reinforcement learning: (1) heightened responses to cues that predicted (affectively) neutral outcomes and (2) increased responses to irrelevant, i.e. unreliable cues. Regarding the first operationalization, blunted differentiation between cues indicating either reinforcement or neutral outcomes were consistently found in schizophrenia patients. Whereas healthy individuals displayed enhanced responses to the reinforced over neutral cues, patients displayed the opposite pattern; increased responses (reaction times, skin conductance as well as midbrain and striatal BOLD responses) to stimuli that were followed by neutral outcomes [29–32]. With regard to the second operationalization, aberrant salience may further be reflected in a tendency or bias towards one over another equally irrelevant stimulus, as defined in the Salience Attribution Test [SAT; 33]. In the SAT, subjects have to speed up their responses to a target to increase their wins. Crucially, the target is preceded by conditioned stimuli with one feature being reliably informative about the following reward (instrumental motivational salience) and another feature being uninformative for predicting reward; being therefore relevant or irrelevant. Here, aberrant salience is reflected by the idiosyncratic bias inside the irrelevant dimension. This quantification of aberrant salience to irrelevant instead of neutral events circumvents instrumental learning deficits reported in schizophrenia [6]. So far, it has revealed mixed results in schizophrenia patients possibly pointing to differential expressions of aberrant salience across the stages of illness. The explicit (= subjective judgment based) aberrant salience measure was increased in first episode patients with delusions and individuals at ultra-high risk for psychosis [33–35] and striatal responses to irrelevant events correlated with positive symptoms [34]. The implicit (= reaction time based) aberrant salience measure was increased in a medicated and rather chronic schizophrenia patients sample [36]. However, other studies using the SAT in patients only found deficits in adaptive salience [37, 38].

In a previous study [39], we found that this idiosyncratic bias inside the irrelevant cue feature does not interfere with adaptive salience attribution that is needed to successfully solve the task. Hypothetically, this may imply that when schizophrenia patients are confronted with cues that are not associated with task-information and that are therefore imprecise they form a bias towards one of these cues in order to resolve this uncertainty. In other words, cues that are irrelevant within a particular (task) framework and that thereby are unreliable in serving instrumental aims may be inherently prone to capture aberrant salience. Thus, explicit task demands should be low in order to create an atmosphere where aberrant salience attribution can arise. A rather implicit task design would further reduce confounds by motivational, cognitive and stress-related deficits known in schizophrenia patients [40–43].

The aim of the current study was to test the idea of aberrant salience as an idiosyncratic bias to subjectively unreliable and thus task-irrelevant information. For that, we used computational modeling in order to assess relevance attribution on the subjective level. While we followed the idea of the SAT of having relevant and irrelevant cue dimensions, we used a more dynamic task design including contingency reversals in order to achieve ongoing learning that is better suited for computational modeling. Further, participants were not instructed to explicitly track contingencies between cues and outcomes in order to keep task demands low.

In the current study, 42 schizophrenia patients and 42 healthy individuals performed an implicit salience paradigm during fMRI [ISP; 39]. In this paradigm, participants had to discriminate between two outcomes (coin/circle) via button press. The outcome could be predicted from preceding graphic cues with dynamically changing contingencies along two distinct features (color and shape). By applying the Bayesian learning framework of the HGF, we used computational modeling to assess individual learning trajectories of these associations. Subjective relevance was formalized as Bayesian precision (as a dynamic reliability measure) and we compared different models, which varied in how subjective relevance affected learning and behavior. We hypothesized that participants would be more surprised by unexpected events and slow down their responses. On the computational level, this was defined via relevance weighted UPEs and we expected their neural correlates to be located in areas previously implicated in salience processing network and/or the striatum. Further, we defined aberrant salience as an idiosyncratic bias towards one unreliable and thus subjectively irrelevant cue feature. We hypothesized that this measure of aberrant salience would be increased in schizophrenia patients.

## Materials and methods

### Ethics statement

All participants gave written informed consent and received monetary compensation as well as the total wins of the task battery. The study was performed in accordance with the Declaration of Helsinki and was approved by the local ethics committee of Charité Universitätsmedizin.

### Participants

In total, 42 schizophrenia patients and a matched healthy control group of 42 individuals participated in this study. Healthy individuals reported no past or present psychiatric disorder according to the SKID-I. Patients were diagnosed with schizophrenia according to the DSM-IV and ICD-10. Psychopathology was assessed using the Positive and Negative Syndrome Scale (PANSS) as well as the subscale for delusions and anhedonia of the Scale for the Assessment of Positive (SAPS) and Negative Symptoms (SANS), respectively (for information on demographics and psychopathology, see Table 1). All patients were on antipsychotic medication (for more details, see **Table A** in the **Supplement**). They were recruited from the inpatient and outpatient units of the Department of Psychiatry and Psychotherapy, Charité-Universitätsmedizin Berlin and the Psychiatric Department of the Schlossparkklinik Berlin. Aberrant salience raw data scores of a partially overlapping sample (37 healthy controls and 34 schizophrenia patients) were reported in a previous publication [39].

**Table 1:** Demographic and clinical characteristics.

	SCHIZOPHRENIA PATIENTS (N = 42)	HEALTHY INDIVIDUALS (N = 42)	STATISTICS
**Gender**	12 females, 30 males	16 females, 26 males	*χ*^*2*^(1) = .355, p = 0.488
**AGE (IN YEARS)**	35.1 (±7.4)	33.5 (±7.8)	*t*(82) = 1, p = 0.321
**VERBAL IQ**	101 (±9.4)	104.9 (±9.4)	*t*(78) = 2.13, p = 0.036
**EHI**	67.4(±53)	69.72 (±48.09)	*t*(77) = .21, p = .837
**DURATION OF ILLNESS (YEARS)**	9.4 (±6)		
**AGE OF ILLNESS ONSET (YEARS)**	25.9 (±7.1)		
**PANSS POSITIVE**	20.7 (±6.7)		
**PANSS NEGATIVE**	22.6 (±7.6)		
**PANSS GENERAL**	41 (±10.9)		
**PANSS TOTAL**	84.5 (±21.5)		
**SAPS DELUSIONS**	21.3 (±10.9)		
**SANS ANHEDONIA**	12.4 (±4.9)		

### Implicit salience paradigm (ISP)

**Fig 1:**
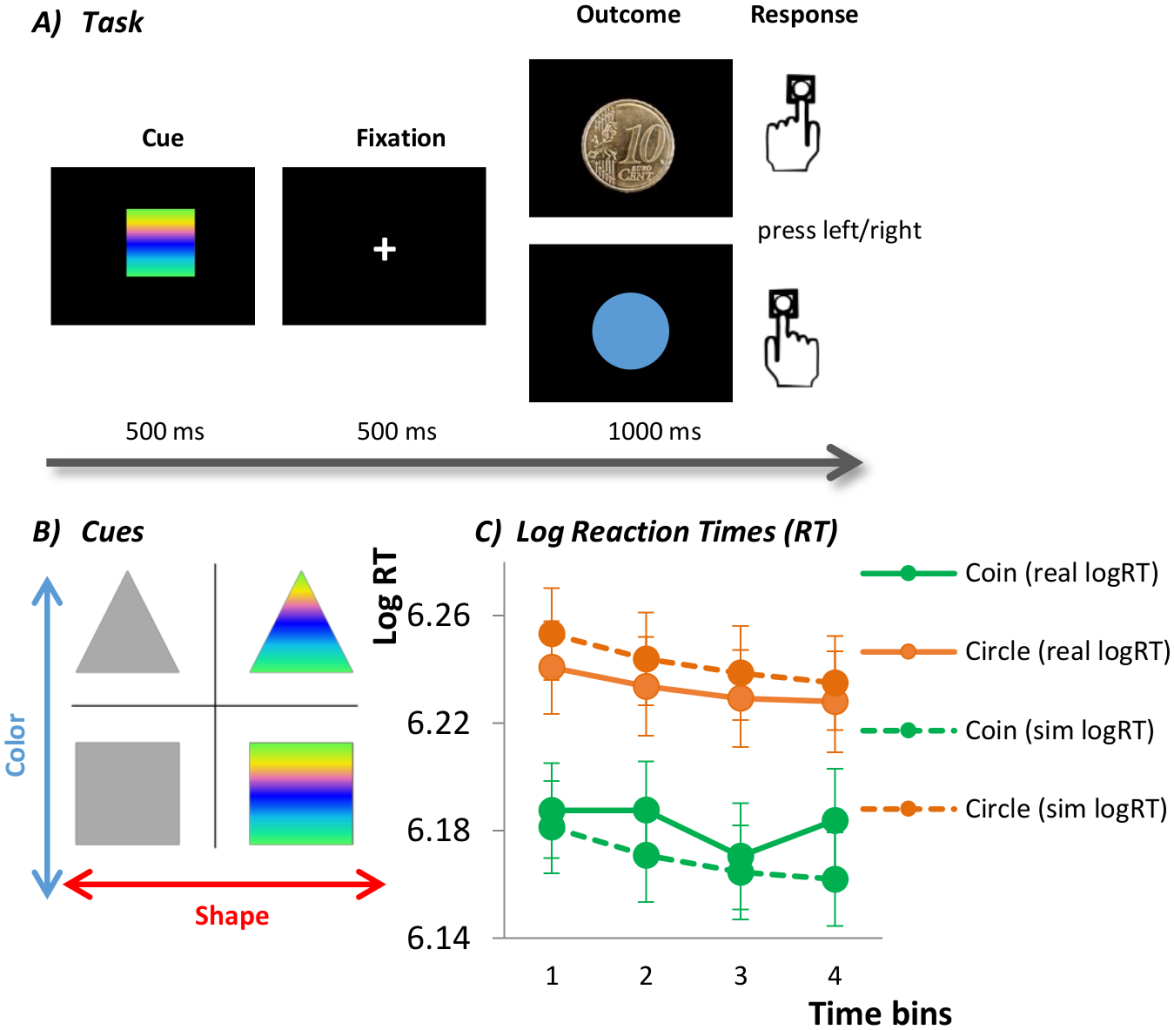
**(A)** Depiction of one ISP trial. Participants see one of four cues (**B**) that is then followed by either a coin (representing reward) or a blue circle (neutral outcome). They have to respond to each outcome via a respective button press. **(B)** Conditioned cues varying in color and shape. **(C)** Log reaction times (logRT) and simulated logRT (dashed lines, based on the best model) for coin (green) and circle (orange) trials. Subjects were faster in coin than in circle trials and speeded up their responses over the course of each block. These effects were also captured by the best fitting model.

**Fig 2:**
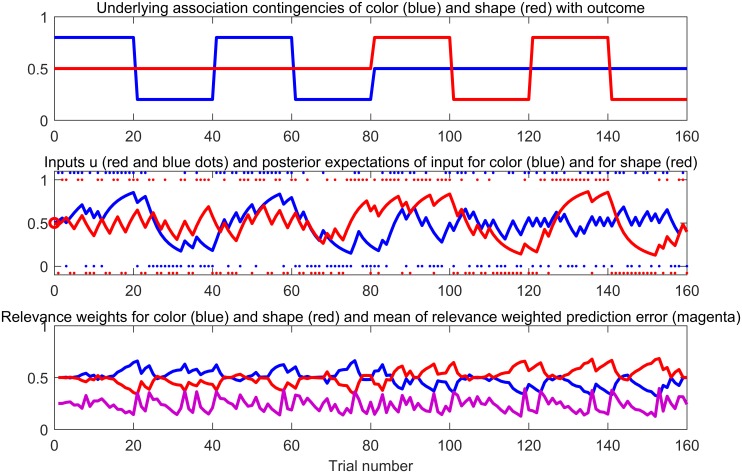
**Upper plot**: Underlying task contingencies between color and outcome (blue) and shape and outcome (red). Note that the direction of the trajectories reflects a tendency towards an association (eg, 1 for [triangle → reward; square →circle] and 0 for the opposite associations) and does not reflect a reward value. In this example, during the first half of the ISP, color is relevant for predicting the outcome, whereas the squares and triangles both predict coin and circle with a probability of 0.5 and are therefore irrelevant. The extradimensional relevance reverses after 80 trials, and shape becomes relevant. Inside the relevant features, the 80% rewarded feature manifestation reverses every 20 trial block. That is to say, if colorful cues are rewarded in 80% of trials (gray cues in 20%) in the beginning, gray cues are followed by coins (colorful cues by a circle) for the next 20 trials. **Middle plot**: One subject’s individual belief trajectories for color (blue) and shape (red) associations with outcome based on the best fitting model (2HGF RelPE+IrrelBias). **Lower plot**: Trajectories that were used to determine individual reaction times in the best response model: Inferred relevance weights (see Eq 5) for color (blue) and shape (red) predictions and the mean relevance weighted prediction error (magenta; see Eq 4.1.2).

This paradigm was explicitly instructed like a target-detection task though implicitly being a learning paradigm where features of neutral stimuli predicted certain outcomes. It consisted of 160 trials where subjects were told to discriminate the outcomes (10 Eurocent coin or blue circle) of each trial. Therefore, their only task was to press a respective button when they saw a coin, versus another button when they saw the blue circle. Subjects were told that they would receive the amount of money they had seen during the task irrespective of whether they had pressed a button or not, though they were encouraged by the experimenters not to miss too many trials because this would impede the analysis. The outcomes were preceded by conditioned stimuli that differed in color and shape: gray or colorful squares or triangles (see Fig 1A and 1B). During the instructions, subjects were told not to pay attention to these stimuli preceding the outcomes. However, prior to scanning, participants were primed with the stimulus features while they were asked to name the color and the shape of each of the four conditioned stimuli. Then, they practiced the outcome detection for 10 trials. In this practice session, all outcomes were preceded by a stimulus that was not presented during the main experiment in the scanner. In the main experiment, the conditioned stimuli predicted the outcome types in a probabilistic manner that reversed during the task. Importantly, only one stimulus feature reliably predicted the outcome (eg, shape). For instance, 80% of all triangles were followed by the coin (20% circle), and 80% of all squares were followed by the blue circle (20% coin). Whether the square or the triangle predicted the coin reversed every 20 trials. In the meantime, the color of the stimuli was irrelevant in predicting the outcome; colorful and gray stimuli were equally followed by coins and circles (50% each). After the first half of the experiment this was reversed, and then the formerly irrelevant feature (here: shape) predicted the outcome, whereas the other feature turned irrelevant (see Fig 2). The relevant dimensions were counterbalanced across participants and coins and neutral outcomes were each displayed in 50% of all trials. In total, the experiment lasted 15 minutes and took place during fMRI scanning. Participants received the amount of coins seen in the experiment (8 Euro).

### Raw data analysis

In our raw data analysis of reaction times, we focused on two aspects: (i) learning of the regularly reversing relevant feature and (ii) aberrant salience towards one manifestation of the irrelevant feature. Extreme reaction times (<150 ms and >1.5 s) were excluded.

For analyses of potential learning, reaction times (in ms) were log transformed to achieve normal distribution required for variance analyses. First, we tested whether subjects learned the cue-outcome contingencies and slowed down their responses if the outcome could not be predicted based on the preceding cues and thus violated their expectation. For that, log reaction times were compared for expected (i.e. trials when the 0.8 rewarded feature was followed by reward and trials when the 0.8 non-rewarded feature was followed by a circle) versus unexpected events (i.e. trials when the 0.8 rewarded feature was followed by the circle and trials when the 0.8 non-rewarded feature was followed by a coin) of the relevant condition in a repeated-measures ANOVA with group as between-subject factor (HC versus patients) and event type as within factor (expected versus unexpected). Second, a change in reaction times over time following contingency changes inside the relevant cue feature was tested using a repeated-measures ANOVA. For all eight blocks with 20 trials each, log-reaction times of the 16 expected events (ie, spanning those 8 trials when the rewarded feature was followed by reward and the 8 trials when the non-rewarded feature was followed by a circle) were grouped for rewarded versus neutral outcome trials (Condition factor) and combined into 4 time bins each, consisting of 2 consecutive trials (Time factor). For analyses targeting responses with regard to different cue features, please see the **Supplement**.

For aberrant salience attribution, aberrant salience scores were calculated as in the previous literature [39], and reflected an individual bias towards one of the two irrelevant cue manifestations based on the ground truth contingencies. For that, the mean reaction times (in ms) to each of the four cue features when they were irrelevant to the task were calculated. Then, aberrant salience was calculated as the absolute difference in reaction times between both manifestations of each condition (eg, square over triangle when shape is irrelevant). Then, these two scores were collapsed across test halves.

### Computational modeling of relevant and irrelevant predictions

We used detailed computational modeling combined with model selection in order to assess the learning mechanisms driving the observed individual behavior. By that we tested if subjects learned the underlying cue-outcome associations and inferred the relevant cue feature. In keeping with our initial definition of relevance, a cue feature should be perceived as more relevant the more precisely it is believed to predict the outcome. In contrast, if the association between cue feature and outcome is very noisy, because the occurrence of reward and circle are equally probable this feature should be perceived as irrelevant. We set up novel response models that postulated slowing down of responses when the expectations were violated. Computationally, a violation was captured by the unsigned PE of each feature. In different models, we compared if unsigned PEs of the different cue features differentially affected behavior based on their current relevance. By the relevance weighted PE, we implemented increased adaptation to surprising events (unsigned PE) predicted by the most relevant cue feature as well as decreased adaptation to the irrelevant cue feature. Thereby, we tested if individuals adapted their behavior less to events when these had proven to be noisy and uninformative in the past. Instead, they would adapt their behavior to those unexpected events that were held to be informative because these may actually signal a real change in the environment (as the contingency changes in our task).

Our modeling analysis was guided by two aims:

1. Modeling two parallel learning processes of predicting the relevant and the irrelevant cue feature in order to compute distinct prediction error trajectories [for a comparable learning model, please see 44]. Here, it is crucial to determine a formal definition of “relevance” since the participants had to dynamically infer which cue feature was relevant for predicting the outcome (even though this was not necessary to solve the task, that is, discriminating between a coin and a circle).
2. Describing the aberrant salience effect to an irrelevant feature as seen in previous analyses [39] on a computational level by finding interindividual differences in model parameters capturing this effect.

We modeled predictions for each of the two cue features, shape and color, in separate HGF learning models so that a trial-by-trial expectation was computed for each feature (learning model). We did not model relevant and irrelevant conditions separately because of two reasons. First, the generative model was supposed to capture the subjects’ learning experience and they were not instructed about the task structure having relevant and irrelevant conditions. Instead they were only primed with the distinction by feature, color and shape, in the practice session. Second, after the first half of the experiment we switched the relevant dimension (e.g. shape to color). Hence, modeling separately for relevance versus irrelevance would have introduced external information on the task structure that was not accessible to subjects and thus not generative. The resulting learning trajectories for shape and color were transformed into trial-by-trial predictors of reaction times (response model).

### Hierarchical Gaussian filter [HGF; 24, 45]

According to the ‘Bayesian brain’ hypothesis [21–23, 46], an agent forms a generative model of the world in part by increasing the precision of predictions (*μ*) to successfully adapt one’s behavior. The HGF offers a generic framework for Bayesian learning on multiple hierarchical levels. Crucially, the belief update at each level is comprised of a lower-level prediction error $\delta_{i-1}^{\left(k\right)}$ that is weighted according to a cross-level precision ratio (Eqs 1 and 2; *i* for learning level, and *k* for trial number). The precision of each level’s prediction ${\hat{\pi }}_{i}^{\left(k\right)}=1/{\hat{\sigma }}_{i}^{\left(k\right)}$ is defined as the inverse variance of the prediction.

$$\Delta \mu_{i}^{k}\propto \frac{{\hat{\pi }}_{i-1}^{\left(k\right)}}{\pi_{i}^{\left(k\right)}}\delta_{i-1}^{\left(k\right)}$$ (1)

$$\Delta \mu_{2}^{k}=\frac{1}{\pi_{2}^{\left(k\right)}}\delta_{1}^{\left(k\right)}$$ (2)

We used a “two branches” version of the HGF for parallel learning of the shape and color associations with the outcome. In our modelling, we focused on the reliability and thus undirected beliefs about associations. Thus, the direction of the learning trajectories (association beliefs ${\hat{\mu }}_{1}^{\left(k\right)}$ and prediction errors *δ*) did not reflect the reward value but an arbitrarily determined relationship between cue feature manifestations and outcomes. A more detailed description of the HGF and its levels can be found in the **Supplement**.

### A novel definition of relevance: First-level precision

Since the core aim of this study was the dissociation between learning about relevant versus irrelevant cue features, the term relevance needs to be defined formally. In terms of the ISP, a cue feature should be perceived as more relevant the more precisely it is believed to predict the outcome. In contrast, if the belief ${\hat{\mu }}_{1}^{\left(k\right)}$ of an association is 0.5 it should be perceived as irrelevant for outcome prediction (because in this case reward and circle will occur with the same probability and cannot be reliably predicted). In the HGF framework, this interpretation of relevance is reflected in the estimated precision of prediction on the first level ${\hat{\pi }}_{1}^{\left(k\right)}$ (see 4).

$${\hat{\pi }}_{1}^{\left(k\right)}=\frac{1}{{\hat{\mu }}_{1}^{\left(k\right)}(1-{\hat{\mu }}_{1}^{\left(k\right)})}$$ (3)

It is a function only of the first-level association prediction ${\hat{\mu }}_{1}^{\left(k\right)}$, which ranges between 0 and 1. ${\hat{\pi }}_{1}^{\left(k\right)}$ has a minimum of 4 for ${\hat{\mu }}_{1}^{\left(k\right)}=0.5$ and increases symmetrically to infinity as ${\hat{\mu }}_{1}^{\left(k\right)}$ approaches 0 or 1. This relevance could affect how beliefs are updated (for this implementation (‘precision feedback’), see the **Supplement**) and/or how learning signals affect behavior, which we implemented in four response models. Our learning model space contained 2-level and 3-level HGFs with and without precision feedback (see Supplement), leading to four different hierarchical learning models: **2HGF, 2HGFprecfb, 3HGF**, and **3HGFprecfb**.

### Response models

Prediction errors relating to the cue feature that is thought to be more relevant might translate more strongly into reaction times, and the aberrant salience effect found in the raw data may be explained as a bias towards one of the irrelevant features on a trial-by-trial basis. Both aspects were formalized in the following four response models. The baseline response model (see Eq 4) postulates that trial-by-trial reaction times are a linear function of the mean of the prediction errors of both features (4.1.1), a constant bias towards one feature manifestation (eg, triangles over squares) and the outcome (eg, slower for circles than for coin trials; 4.2.1). The four cue manifestation vectors *m* reflect whether the respective manifestations were displayed for every trial (eg, 1 for triangles and 0 for squares in *m*^*triangle*^).

$$\begin{array}{lll} \text{log}\left(\text{rt}\right) & =\beta_{0} & \\ & +\beta_{1}\frac{\left|\delta_{1}^{\left(color\right)}\right|+\left|\delta_{1}^{\left(shape\right)}\right|}{2} & |\left(4.1.1\right)\text{Mean}\;\text{of}\;\text{first-level}\;\text{prediction}\;\text{errors}\\ & -(\beta_{2}*m^{colorful}+\beta_{3}*m^{grey})-(\beta_{4}*m^{square}+\beta_{5}*m^{triangle}) & |\left(4.2.1\right)\text{Manifestation}\;\text{bias}\\ & +\beta_{6}\left(Outcome\right) & |\text{Reward}\;\text{effect}\;\text{i}.\text{e}.\;\text{faster}\;\text{response}\;\text{towards}\;\text{reward}\;\left(\text{Outcome}:\;\text{Circle}=1,\;\text{Coin}=0\right)\\ & +\zeta & |\text{Gaussian}\;\text{Noise} \end{array}$$ (4)

This response model was modified in three ways. In a first modification (4.1.2), the absolute prediction errors of each feature were weighted according to their respective relevance weight (*rel*; 5). The latter is formalized as the relative amount of each feature’s precision given the overall precisions of both features.

$$\begin{array}{l} +\beta_{1}\frac{rel^{\left(color\right)}*\left|\delta_{1}^{\left(color\right)}\right|+rel^{\left(shape\right)}*\left|\delta_{1}^{\left(shape\right)}\right|}{2}+\left(\ldots \right) \end{array}$$ (4.1.2)

$$\left(\ldots \right)-irrel^{color}\;(\beta_{2}*m^{colorful}+\beta_{3}*m^{grey})-irrel^{shape}(\beta_{4}*m^{square}+\beta_{5}*m^{triangle})+\left(\ldots \right)$$ (4.2.2)

$$rel^{color}=\frac{{\hat{\pi }}_{1}^{\left(color\right)}}{{\hat{\pi }}_{1}^{\left(color\right)}+{\hat{\pi }}_{1}^{\left(shape\right)}}=irrel^{shape};\;rel^{shape}=\frac{{\hat{\pi }}_{1}^{\left(shape\right)}}{{\hat{\pi }}_{1}^{\left(color\right)}+{\hat{\pi }}_{1}^{\left(shape\right)}}=irrel^{color};$$ (5)

In a second modification and in line with the aberrant salience effect, the constant bias was weighted according to its feature’s *irrelevance* (*irrel*; see Eq 4.2.2). The irrelevance weight of one feature was defined as the relevance weight of the opposing feature (see Eq 5). From the four cue feature parameters (*β*_2_, …, *β*_5_) one composite parameter was calculated in two steps. First, the individual absolute differences between the *β*_2_ and *β*_3_ for irrelevance weighted manifestations within color (and between the *β*_4_ and *β*_5_ for shape manifestations) were calculated. Then, they were collapsed across color and shape to achieve one parameter *β*_*irrelevance*_ capturing the bias towards one cue feature manifestation that increased with subjective irrelevance.

Thus, we compared four different response models: the baseline model (Eq 4), the baseline model with relevance weighted absolute prediction errors (Eq 4 with modification term 4.1.2; RelPE), the baseline model with only the irrelevance bias (Eq 4 with modification term 4.2.2; IrrelBias), and a full model with both modifications (Eq 4 with modification terms 4.1.2 and 4.2.2; RelPE+IrrelBias). This led to a total model space of 16 model combinations (see **Figure S1**).

### Model fitting and Bayesian model comparison

All models were fitted using the HGF toolbox 4.15 [24, 45] as part of TNU Algorithms for Psychiatry-Advancing Science (TAPAS, http://www.translationalneuromodeling.org/tapas/). For optimization, a quasi-Newton optimization algorithm was applied. We used random-effects Bayesian Model Selection [BMS, spm_BMS in SPM12, www.fil.ion.ucl.uk/spm; 47] for each subject’s and each model’s negative free energy (as an approximation to log-model evidence) in order to identify which of the competing models best explained the subjects’ response time data. BMS takes into account accuracy of each model and also penalizes for complexity. It accounts for heterogeneity across subjects and treats each model as a random variable in the population. We report protected exceedance probabilities for each model (PXP) and posterior probabilities (PP) as well as exceedance probabilities (XP) for model families (HGF vs. HGFprecfb; 2HGF vs. 3HGF; BL vs. RelPE vs. IrrelBias vs. RelPE+IrrelBias). The XP describes the relative probability that the model would better replicate the data in comparison with the other models. The PXP that governed our model selection protects against the ‘null’ possibility that there are no differences in the likelihood of models across the population.

### Simulation analyses

Based on the estimated individual parameters from the best-fitting model, we simulated trial-by-trial reaction time data. In addition to the Bayesian model comparison, we tested the model’s credibility by carrying out the same analyses as in the raw data section on the simulated data ((1) Expectedness*Group ANOVA, (2) Condition*Time*Group ANOVA, (3) ‘ground truth’ aberrant salience group differences). Using this approach, we checked whether the model was capable of reproducing the meaningful effects and group differences that were evident in the data.

### Functional magnetic resonance imaging

For single and group statistics, an event-related analysis was applied using the general linear model (GLM) approach as implemented in SPM12 (http://www.fil.ion.ucl.ac.uk/spm/software/spm12/). On the single subject level, the outcome onsets were convolved with the hemodynamic response function and its temporal derivative. As parametric modulator, the mean of the relevance weighted absolute prediction errors of the best fitting model (2HGF-RelPe+IrrelBias) was introduced, representing how unexpected and salient the observed outcome was based on the subjects’ learned expectations about the two cue features. Regressors of no interest were no response trials, trials with reaction times>1.5 seconds and <150 milliseconds, realignment parameters with their first temporal derivative of translational movement, and one regressor for scans with >1mm scan-to-scan movement. For random effects group-level analysis, the individual contrast images for mean relevance weighted PEs were used in a two-sample t-test for between-group comparisons (controls vs. patients). Explorative analysis probed the association between model parameter *β*_*irrelevance*_ and neural relevance weighted PE signals in schizophrenia patients. Hence, interindividual *β*_*irrelevance*_ scores were introduced as a covariate in a one-sample t-test using the Mean relevance weighted PE contrast. Results are reported using FWE correction at the voxel level across the whole brain. Based on our hypothesis concerning activations in areas previously shown to code salience such as the ACC and insula, namely the so-called salience network [8–11], and the nucleus accumbens, we applied small volume correction at the voxel level for the respective bilateral anatomical masks derived from the WFU PickAtlas (http://fmri.wfubmc.edu/software/pickatlas). Thus, three VOIs were used for small volume correction at *p*_FWE_<0.05 and we indicate which results survive Bonferroni correction for three tests.

## Results

### Raw data analysis

Participants displayed increased log reaction times observed for probabilistic (unexpected) events compared to non-probabilistic (expected) events (Main effect of event type: *F*(1,82) = 5.9, *p* = 0.018, interaction event type*group *F*(1,82) = 0.02, *p*>0.8). The Reward*Time*Group ANOVA revealed that reaction times differed significantly between coin and circle (main effect Condition: *F*(1, 82) = 22.78, *p* < .001, see Fig 1C) and showed a trend-wise decrease following a reversal (main effect Time: *F*(3, 246) = 2.6, *p* = .053). Participants took around 8 trials to decrease their reaction times after slowing down following a reversal in contingencies (RT difference first time bin (trials 1–4) vs. third time bin (trials 8–12), *t* = 2.72, *p* = 0.048, Bonferroni corrected). Groups did not significantly differ in their reaction times (main effect of Group *p*>.2). The aberrant salience scores calculated based on the ground truth contingencies differed significantly from zero in both groups (mean (SD) for HC = 17.98 (10.88), *t*(41) = 10.7, *p* < .001; for Sz = 22.95 (14.46), *t*(41) = 10.3, *p* < .001). Schizophrenia patients displayed increased aberrant salience scores compared to healthy individuals (Welch’s *F*(1, 76.156) = 3.2, *p* = 0.04, one-tailed based on our a priori hypothesis).

### Bayesian model comparison

Across all subjects, the two-level HGF with the full response model was the best fitting model (PP = 0.3755; PXP = 0.5155; see Table 2). Among learning models there was clear evidence against the precision feedback model (PP_HGF_ = .968; PP_HGFprecfb_ = .032; exceedance probability XP_HGF_ = 1), while there was only a very subtle advantage for two-level compared to three-level models (PP_2HGF_ = .551; PP_3HGF_ = .449; XP_2HGF_ = .551; XP_3HGF_ = .449). Concerning the response models, the full response model using the mean relevance weighted prediction error and the irrelevance weighted bias clearly explained the data best (PP_BL_ = .015; PP_irrelBias_ = .037; PP_relPE_ = .017; PP_relPE+irrelBias_ = .93; XP_relPE+irrelBias_ = 1). Therefore, we decided to do our fMRI analyses with the 2HGF model and the best response model relPE+irrelBias.

**Table 2:** Bayesian model comparison of the 16 model combinations.

		2HGF-BL	2HGF-relPE	2HGF-irrelBias	2HGF-full	2HGFprecfb-BL	2HGFprecfb-relPE	2HGFprecfb-irrelBias	2HGFprecfb-full	3HGF-BL	3HGF-relPE	3HGF-irrelBias	3HGF-full	3HGFprecfb-BL	3HGFprecfb-relPE	3HGFprecfb-irrelBias	3HGFprecfb-full
**Full sample**	**PP**	0.012	0.013	0.023	**0.375**	0.012	0.013	0.017	0.036	0.012	0.013	0.023	0.372	0.012	0.013	0.021	0.035
	**XP**	0	0	0	**0.515**	0	0	0	0	0	0	0	0.485	0	0	0	0
	**PXP**	0	0	0	**0.515**	0	0	0	0	0	0	0	0.485	0	0	0	0
**Only HC**	**PP**	0.02	0.022	0.037	**0.304**	0.02	0.021	0.027	0.049	0.02	0.022	0.037	0.303	0.02	0.021	0.028	0.047
	**XP**	0	0	0	**0.503**	0	0	0	0	0	0	0	0.497	0	0	0	0
	**PXP**	0.001	0.001	0.001	**0.497**	0.001	0.001	0.001	0.001	0.001	0.001	0.001	0.49	0.001	0.001	0.001	0.001
**Only Sz**	**PP**	0.021	0.023	0.038	**0.278**	0.02	0.022	0.029	0.064	0.021	0.023	0.038	0.276	0.02	0.022	0.041	0.062
	**XP**	0	0	0	**0.505**	0	0	0	0	0	0	0	0.494	0	0	0	0
	**PXP**	0.01	0.01	0.01	**0.434**	0.01	0.01	0.01	0.01	0.01	0.01	0.01	0.424	0.01	0.01	0.01	0.01

HC = healthy controls; Sz = schizophrenia patients; PP = Posterior probability; XP = exceedance probability; PXP = protected exceedance probability; BL = Baseline; relPE = relevance weighted prediction error; irrelBias = irrelevance weighted bias; full = full response model incl. relevance weighted PE and irrelevance bias;

### Model checking by data simulation

We repeated the same analyses as for the raw reaction time data for the simulated log RTs based on the best fitting model. The ANOVAs revealed similar behavioral effects: faster responses for expected than for unexpected events (*F*(1,82) = 296.8, *p* < .001), faster responses for coins than for circles (*F*(1,82) = 118.4, *p* < .001), as well as faster reaction times across time bins (*F*(1.6, 133.3) = 216.3, *p* < .001; see Fig 1C). The aberrant salience difference score was again significantly increased in schizophrenia patients (Mean = 14.4, SD = 8.2) compared to healthy individuals (Mean = 10.2, SD = 5.9) (Welch’s *F*(1, 74.6) = 7.2, *p* = .009) and correlated with the aberrant salience score from the raw data (*Pearson’s r* = 0.637, *p*<0.001). In line with that, *β*_*irrelevance*_ reflecting the model based subjective bias towards irrelevant events differed significantly from zero in both groups (HC: *t*(41) = 12.6, *p*<0.001; Sz: *t*(41) = 12.1, *p*<0.001) and correlated with the aberrant salience difference score from the raw data analysis (HC: *ρ* = 0.523, *p*<0.001; Sz: *ρ* = 0.612, *p*<0.001).

### Response model parameter differences

**Fig 3:**
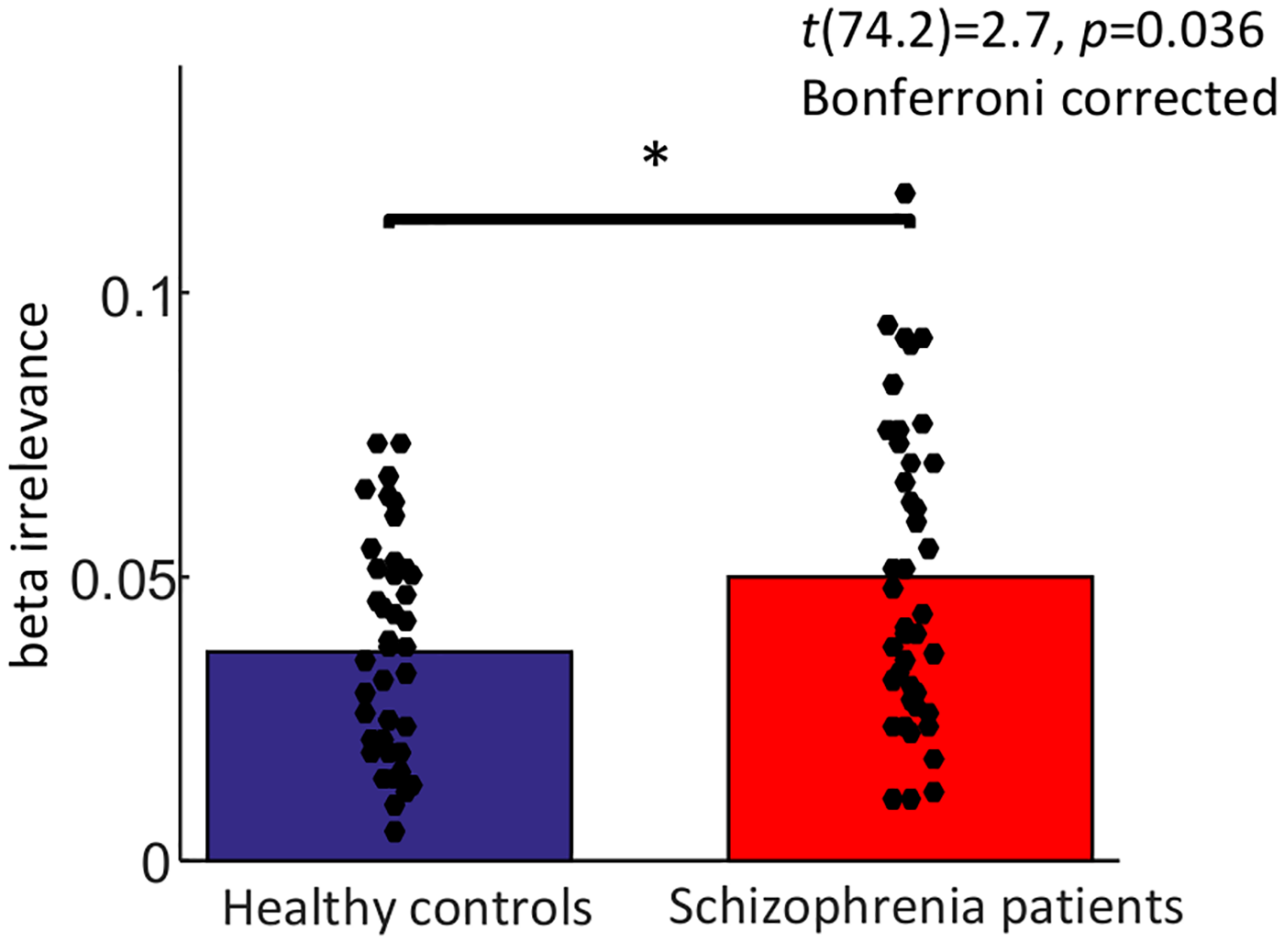
Group means and individual values of the model parameter *β_irrelevance_* that captures the idiosyncratic bias to one out of the two subjectively irrelevant cue features.

We tested for group differences on three individual response parameters that weighted the influence of the relevance weighted prediction error (*β*_1_), the outcome (*β*_6_), and the irrelevance bias (*β*_*irrelevance*_) on reaction times. Schizophrenia patients showed an increased bias towards one of two equally irrelevant cue features as indicated by increased *β*_*irrelevance*_ values (*t*(74.2) = 2.7, *p* = 0.036, corrected for multiple comparisons, see Fig 3). Both groups did not differ on *β*_1_ and *β*_6_ (*p*>0.7). For all fitted model parameters, see Table 3.

**Table 3:** Mean (SD) free parameter estimates of the best fitting model 2HGF-relPE+irrelPE.

Free parameters	Schizophrenia patients	Healthy individuals
$\sigma_{2}^{0}$	.05 (±.0007)	.05 (±.0008)
*ω*	-1.97(±.09)	-1.98(±.08)
*β*_0_	6.27 (±.15)	6.22 (±.18)
*β*_1_	.21 (±.10)	.20 (±.10)
*β*_*irrelevance*_	.050 (±.027)	.037(±.02)
*β*_6_	.07 (±.06)	.08 (±.06)
ζ	.04 (±.22)	.03 (±.01)

### Correlation analyses

In an explorative approach, we investigated how the response model parameter *β*_*irrelevance*_ related to psychopathology using Spearman’s correlations within groups. In schizophrenia patients, *β*_*irrelevance*_ was associated with an increased negative symptoms score from the PANSS (*ρ* = 0.334, *p* = 0.031) but there was no significant correlation with the other PANSS scores (all *p*-values>0.2; except *p* = 0.11 for total PANSS score).

### Model based fMRI

**Fig 4:**
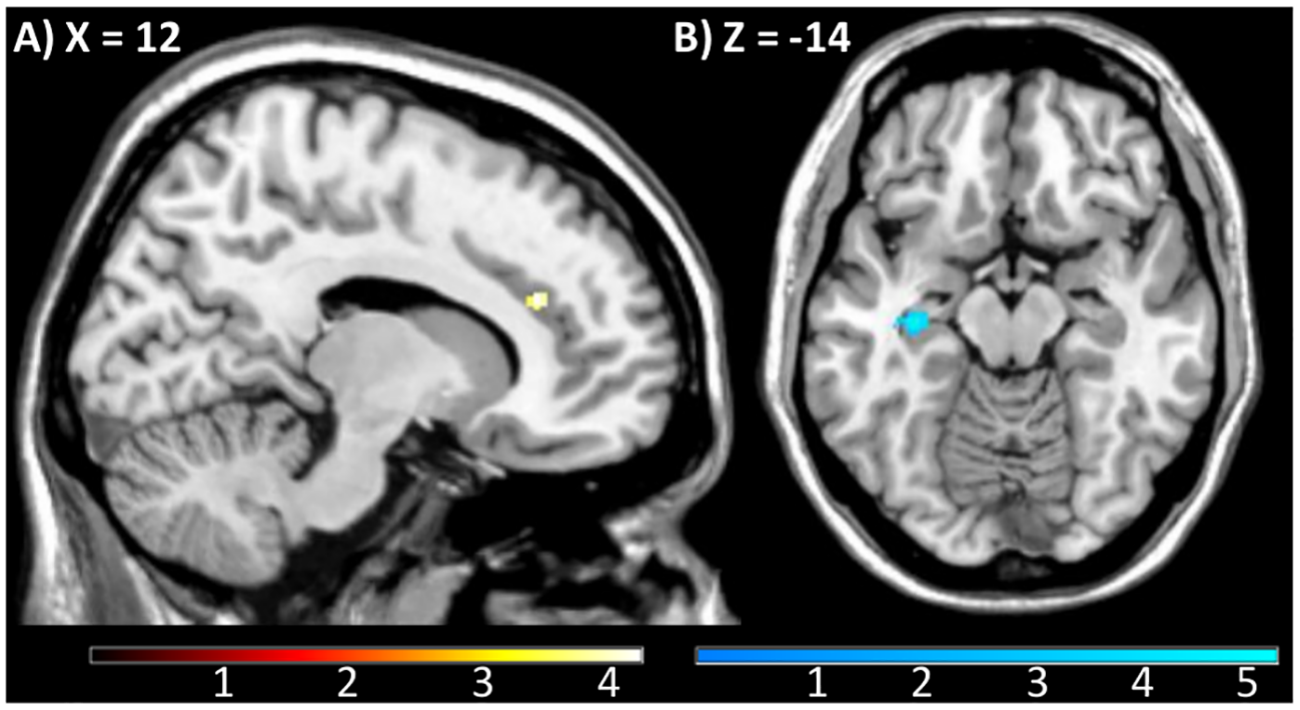
(A) Across all participants, relevance weighted PEs correlate with increased BOLD response in the right ACC ([12 32 22],], t(1,74) = 4.22, p_SVC for ACC VOI_ = 0.032) and (B) decreased left hippocampus response ([-32–18–14], all plots displayed at p<0.001, k>10).

Across all participants, the relevance weighted prediction error correlated with increased BOLD response in the anterior cingulate cortex ([12 32 22], *t*(74) = 4.2, *p*_SVC for ACC VOI_ = 0.032, *p*_B corr_ = 0.096, see Fig 4A). A negative correlation with relPE was observed in the left hippocampus ([-32–18–14], *t*(74) = 5.4, *p*_FWE whole brain_ = 0.041, see Fig 4C). There was no group difference in relevance weighted PE response in any of the VOIs nor at the whole brain level.

**Fig 5:**
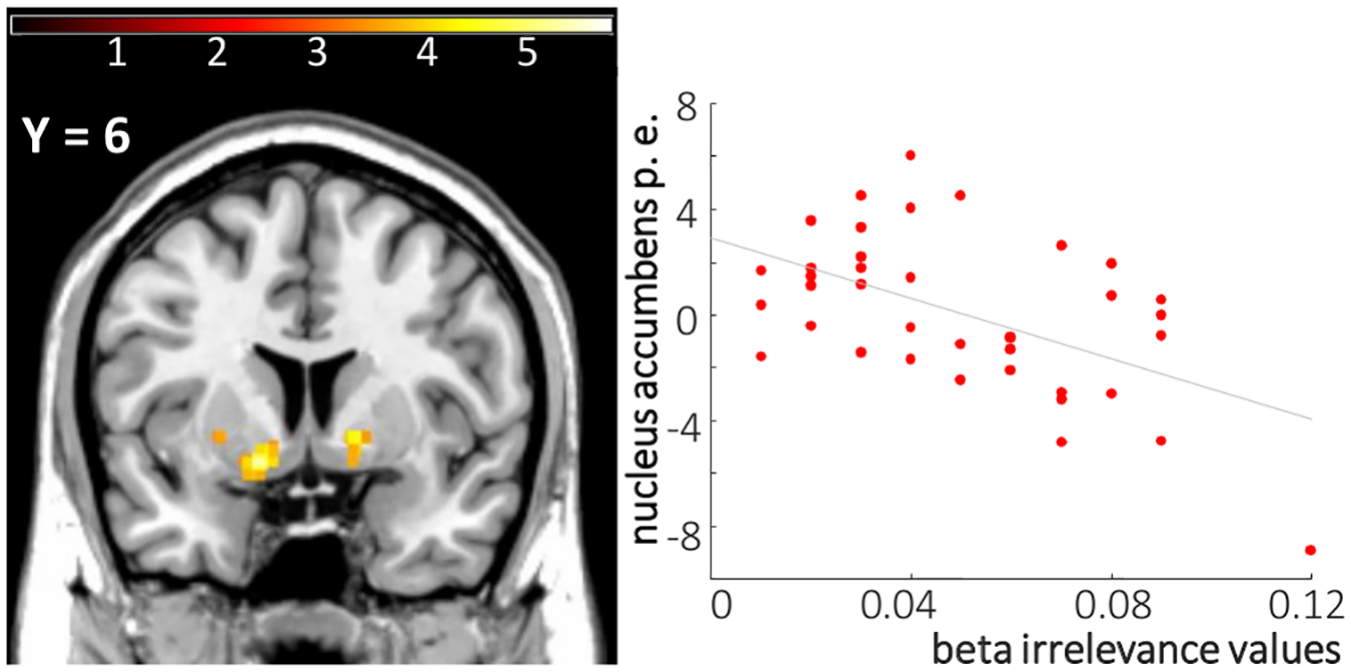
In schizophrenia patients, the tendency towards an irrelevant cue feature (β_irrelevance_) correlated with decreased response to relevance weighted PEs in the bilateral nucleus accumbens (p. e. for parameter estimates; [-14 4–10] and [14 6–8], displayed at p<0.001, k>10).

In order to probe the associations between model derived parameters and brain responses in patients, we focused on the model parameter *β*_*irrelevance*_ that was increased in schizophrenia patients and related to psychopatholgy. In schizophrenia patients, there was an inverse correlation between *β*_*irrelevance*_ values and relPE related bilateral nucleus accumbens response ([-14 4–10], *t*(36) = 5.21, *p*_SVC for nucleus accumbens_<0.001; *p*_Bonferroni corr_ = 0.001; [14 6–8], *t*(36) = 3.6, *p*_SVC for nucleus accumbens_ = 0.019, *p*_Bonferroni corr_ = 0.057, see Fig 5).

## Discussion

In the current study, we established a novel definition of subjective relevance based on Bayesian precision of predictions. This computational mechanism was involved in implicit learning about multidimensional and changing environments, as well as in aberrant salience attribution in schizophrenia. To our knowledge, our study stands alone in investigating subjective beliefs during implicit learning in a dynamic appetitive Pavlovian conditioning task. We had three main findings: 1) Both groups learned the underlying associations equally well but patients showed more aberrant salience in terms of a bias towards task-irrelevant features; 2) in all participants unexpected outcomes as indicated by high relevance-weighted unsigned prediction errors were associated with increased dorsal ACC BOLD signal as part of the so-called salience network; and 3) heightened aberrant salience in patients in terms of a bias towards the currently task-irrelevant stimulus feature was associated with a lower neural salience signal in the nucleus accumbens and higher negative symptom severity.

On the behavioral level, patients and controls both performed the target detection task with high accuracy and responded faster to the rewarding coin stimulus compared to the neutral circle. These two findings suggested that participants of both groups were engaged in the simple task of pressing one button upon seeing a coin and another for a circle, which had obviously minimal cognitive demands. We found that schizophrenia patients and healthy individuals seemed to use the preceding cues to speed up during the outcome discrimination task.

Subjects were faster for expected than for unexpected events in terms of ground truth probabilistic contingencies. Detailed computational modeling combined with model selection revealed that participants learned and used the underlying cue-outcome associations and could determine the currently relevant feature. Thus, participants discriminated faster if the outcome was predicted based on the preceding cues but slowed down if the observed outcome violated the expectation, which was formalized in the relevance weighted PE. Responses to the most relevant cue feature were increased, whereas reactions to the irrelevant feature were decreased as implemented in the best-fitting response model. Hence, subjects adapted more to those unexpected events that were thought to be reliable and that thus signaled actual changes in the environment. Correspondingly, they downregulated responses to such information that was thought to be noisy and uninformative. To conclude, in our task the relevance weighted unsigned PE can be interpreted as subjective informative surprise that leads to subtle adaptation in behavior even in the absence of instrumental need.

While relevance weights scaled the influence of prediction errors on reaction times, that is, behavioral adaptation, we had no evidence that updating of association beliefs was increased by the subjective relevance of a cue feature beyond the HGF implementation of a cross level precision ratio [24, 45]. This may only hold for our implicit and dynamic paradigm and seems to be different when subjects are explicitly instructed to find out the steadily relevant aspect of a multidimensional learning cue [18].

The relevance weighted unsigned PE correlated with BOLD responses in the salience network comprising the dorsal ACC. This is in line with the theory of proximal salience, which proposes that activity of ACC and insula regulates higher order processing of external stimuli [16]. Especially the dorsal ACC/medial prefrontal cortex has been reported to respond to unexpectedness regardless of valence [48, 49]. According to the response-outcome theory [50], the dorsal ACC is crucial for detecting discrepancies between expectations and outcomes and thereby drives attentional and behavioral reallocation. Further studies that elaborated how individuals use those unexpectedness signals highlighted the ACC’s function in belief updating [51] and in predicting future cognitive load based on previous experience [14]. This can be related to the relevance weighted unsigned PE signal in our study that also comprised estimates of prior reliability of a cue feature. In line with our results, a recent study also used computational modeling of Bayesian conflict learning and reported similar UPE correlates in the dorsal ACC [12]. On the other hand, the hippocampus showed an opposite pattern in our study: high hippocampus activation was associated with low relevance weighted UPE. This stands in contrast to previous findings and theories describing hippocampal activation during mismatching events [for a review see 52]. In our task, low relevance weighted UPE occurred when the observed outcome was not surprising and would therefore be mostly present at the end of each block, after constantly changing contingencies have been learned. In other words, expected events elicit stronger BOLD response in the hippocampus which might indicate higher-order processes related to contextualizing and memorizing these learned contingencies [53, 54].

Although participants were not incentivized for target detection, both groups performed well and no group differences emerged for learning contingencies indicating that patients and controls both used the cues for behavioral adaptation. Contrarily, there is sound evidence for learning deficits in schizophrenia patients mostly in more explicit and instrumental tasks [55–58]. Our study assessed implicit learning which might have led to the subtle behavioral and neural effects. In addition, the reversals inside the relevant condition appeared every 20 trials. Presumably, fewer reversals with longer stable periods for learning the implicit associations might have led to stronger task effects. Switching the relevant condition in the middle of the experiment did not affect learning significantly (please see Supplement), though this seems to be different in tasks where such shifts happen more often and are explicitly instructed as during set-shifting. During these paradigms, schizophrenia patients are impaired [55]. Hence, whereas we focused on the rather implicit and prediction error driven learning about relevance with our paradigm and model space, schizophrenia patients might show more pronounced deficits when explicit reasoning about the structure of the task is required. It has been shown that when healthy individuals were explicitly asked to find out the relevant cue dimension they used explicit strategies reflecting the assumed underlying task structure [59]. Thus, group differences between schizophrenia patients and healthy individuals concerning the detection of relevance (shifting) might be better detectable and more pronounced in such more complex learning paradigms probing deliberative decisions that rely on the use of explicit task knowledge.

In keeping with previous results in a partially overlapping sample [39], schizophrenia patients displayed an increased bias for one of two equally task-irrelevant cue features as formalized using the ground truth contingencies of the task. This is in line with previous studies that reported increased responses in schizophrenia patients to neutral [29–32, 60] or unreliable [33] information. We further elucidated this bias using computational modeling that took into account only subjective and dynamic relevance estimates. The response parameter capturing this irrelevance bias was increased in patients indicating that they attributed more aberrant salience to cues when they were subjectively irrelevant and thus thought to be less informative with regard to the task. This aberrant salience bias was associated with decreased striatal activation during relevance weighted PE signaling. Though this finding has to be treated with caution, since this region did not display a task effect per se, it might show that patients experiencing more idiosyncratic and task-unrelated saliency also showed a reduced striatal processing of relevant information. There is meta-analytic evidence for decreased striatal responses in schizophrenia patients to reward-predicting cues and rewarding feedback [61]. Also, ultra-high risk subjects who decreased their unusual thought content after treatment showed an amelioration of striatal response to relevant and reinforced stimuli in the SAT [35]. These studies focused on striatal reward anticipation, whereas the relevance weighted PE in our study was undirected, i.e. carried no information about reward, only about associability strength and the respective surprise when these associations were violated in both directions, good or bad. This striatal PE coding is in line with a recent study on explicit reasoning that elegantly decorrelated reward PEs from Bayesian surprise and the authors found that the latter was more strongly associated with striatal response [59]. Taken together, the processing of relevant (not only rewarding) and the bias towards irrelevant information seem to be interfering phenomena. We cannot make any claims on causality here and would argue that bidirectional influences are plausible and may appear at the same time. A possible interpretation may be that not being able to figure out the correct (e.g. rewarding) cues for behavioral adaptation may cause a compensatory clinging to random cues as seen in the aberrant salience bias to irrelevant events in our study.

Note that this aberrant salience definition which is based on the SAT literature [33, 35] targets increased responses to task-irrelevant and not to neutral events as often used in previous Pavlovian studies [30, 31, 62]. This different operationalization of aberrant salience could be important for associations with psychopathology. One study reported increased BOLD response towards cues reliably indicating neutral outcomes to be associated with positive symptoms [30]. Our aberrant salience measure to events that were learned to be uninformative of *any* outcome relates to negative symptoms. In the same vein, orientating behavior to unreliable, and thus irrelevant stimuli was proposed to underlie negative symptom formation [5]. Taken together, a biased focus on uninformative, irrelevant events by possibly limiting attention to relevant events relates to increased negative symptoms. Roiser and colleagues found a similar association between (explicit) aberrant salience to irrelevant events and negative symptoms in patients as well as with anhedonia in healthy individuals [33, 63]. They interpreted this to result from ‘false negatives’ in phasic dopamine signaling to contextually relevant events contributing to reduced processing of reinforcing stimuli [33]. This view is supported by an animal study reporting decreased striatal dopamine transients to relevant, reward-predicting stimuli following amphetamine administration [64]. In the same vein, aberrant salience was related to increased tonic dopamine synthesis capacity and reduced responses to relevant events in the striatum in healthy individuals [65]. Transferred to our findings, processing unreliable information in schizophrenia patients may increase processing of irrelevant as well as decrease processing of relevant events while contributing to both, positive and negative symptoms [4, 5]. Our association between the aberrant salience (irrelevance) bias and negative symptoms was found in a chronic patient sample on stable antipsychotic medication showing both negative and positive symptoms.

Taken together, we provide evidence that schizophrenia patients show a bias towards irrelevant stimuli when confronted with an uncertain and changing environment. Future longitudinal studies should examine the time-wise formation of this bias as well as the process of relevance detection and their respective associations with psychopathology.

Several limitations of our study need to be addressed. First, by keeping the contingency structure implicit, it possibly led to a high variance between subjects in how to solve the paradigm. We modeled reaction times and although there are notable exceptions [66–69], modeling of choice data is more widely used. For reaction time based analysis, unlike choice-based analysis, there is no clear absolute model fit to compare against as in previous studies [56, 57]. Second, because learning was not necessary for task performance, the implicit behavioral and neural learning effects were both very subtle and might need larger samples for the detection of group differences. Third, the two different cue features were initially chosen to be easily dissociable resulting in differences regarding their perceptual characteristics and presumably their saliency. As alterations of visual perception have been reported in schizophrenia patients, patients might have processed the cue features differently compared to controls [70]. However, in our supplementary analyses we neither found evidence that the cue features were learned differently nor that cue features were processed differently between groups. Fourth, with our current paradigm we cannot disentangle the saliency of cues from their rewarding valence since we did not include a punishment condition. Finally, future studies of learning about multidimensional information in schizophrenia should include additional methods to detect relevance attribution, such as skin conductance response [30], eye-tracking, or MVPA [18].

In sum, we give a novel computational account of the use of subjective relevance estimates in implicit learning that is based on Bayesian precision. Furthermore, we provide quantitative, model-based evidence of an impairment in the formation and/or use of relevance estimates associated with schizophrenia. In a task probing the implicit learning of multidimensional and dynamic associations, relevance detection and neural learning correlates in the ACC seem to be intact in patients with schizophrenia, but aberrant salience to subjectively irrelevant events was increased in patients and related to negative symptoms and reduced striatal response to salient events. Our findings suggest that individual beliefs about relevance can be inferred from computational models and highlight the importance of relevance detection to complex environmental stimuli.

## Supporting information

S1 Text Supplementary material. The supplement contains the following analyses and results: additional information on the ISP and raw data analyses of potential stimulus feature effects, additional information on the HGF and the HGF-precision feedback learning model, overview of the model space, fMRI preprocessing, and tables of the priors of learning and response models.
(DOCX)

## References

[1] Heinz A. Dopaminergic dysfunction in alcoholism and schizophrenia—psychopathological and behavioral correlates. European psychiatry: the journal of the Association of European Psychiatrists. 2002;17(1):9–16. . 11918987 10.1016/s0924-9338(02)00628-4

[2] Kapur S. Psychosis as a state of aberrant salience: a framework linking biology, phenomenology, and pharmacology in schizophrenia. The American journal of psychiatry. 2003;160(1):13–23. 10.1176/appi.ajp.160.1.13 . 12505794

[3] Winton-Brown TT, Fusar-Poli P, Ungless MA, Howes OD. Dopaminergic basis of salience dysregulation in psychosis. Trends Neurosci. 2014;37(2):85–94. 10.1016/j.tins.2013.11.003 . 24388426

[4] Maia TV, Frank MJ. An Integrative Perspective on the Role of Dopamine in Schizophrenia. Biological psychiatry. 2017;81(1):52–66. 10.1016/j.biopsych.2016.05.021 . 27452791 PMC5486232

[5] Corlett PR, Honey GD, Fletcher PC. Prediction error, ketamine and psychosis: An updated model. J Psychopharmacol. 2016;30(11):1145–55. 10.1177/0269881116650087 . 27226342 PMC5105325

[6] Deserno L, Boehme R, Heinz A, Schlagenhauf F. Reinforcement learning and dopamine in schizophrenia: dimensions of symptoms or specific features of a disease group? Frontiers in psychiatry. 2013;4:172 10.3389/fpsyt.2013.00172 . 24391603 PMC3870301

[7] Diederen KM, Spencer T, Vestergaard MD, Fletcher PC, Schultz W. Adaptive Prediction Error Coding in the Human Midbrain and Striatum Facilitates Behavioral Adaptation and Learning Efficiency. Neuron. 2016;90(5):1127–38. 10.1016/j.neuron.2016.04.019 . 27181060 PMC4893165

[8] White TP, Joseph V, Francis ST, Liddle PF. Aberrant salience network (bilateral insula and anterior cingulate cortex) connectivity during information processing in schizophrenia. Schizophrenia research. 2010;123(2–3):105–15. 10.1016/j.schres.2010.07.020 . 20724114

[9] Seeley WW, Menon V, Schatzberg AF, Keller J, Glover GH, Kenna H, et al Dissociable intrinsic connectivity networks for salience processing and executive control. The Journal of neuroscience: the official journal of the Society for Neuroscience. 2007;27(9):2349–56. 10.1523/JNEUROSCI.5587-06.2007 . 17329432 PMC2680293

[10] Peters SK, Dunlop K, Downar J. Cortico-Striatal-Thalamic Loop Circuits of the Salience Network: A Central Pathway in Psychiatric Disease and Treatment. Front Syst Neurosci. 2016;10:104 10.3389/fnsys.2016.00104 . 28082874 PMC5187454

[11] Menon V, Uddin LQ. Saliency, switching, attention and control: a network model of insula function. Brain Struct Funct. 2010;214(5–6):655–67. 10.1007/s00429-010-0262-0 . 20512370 PMC2899886

[12] Manza P, Hu S, Ide JS, Farr OM, Zhang S, Leung HC, et al The effects of methylphenidate on cerebral responses to conflict anticipation and unsigned prediction error in a stop-signal task. J Psychopharmacol. 2016;30(3):283–93. 10.1177/0269881115625102 . 26755547 PMC4837899

[13] Cavanagh JF, Figueroa CM, Cohen MX, Frank MJ. Frontal theta reflects uncertainty and unexpectedness during exploration and exploitation. Cereb Cortex. 2012;22(11):2575–86. 10.1093/cercor/bhr332 . 22120491 PMC4296208

[14] Grundy JG, Shedden JM. Support for a history-dependent predictive model of dACC activity in producing the bivalency effect: an event-related potential study. Neuropsychologia. 2014;57:166–78. 10.1016/j.neuropsychologia.2014.03.008 . 24686093

[15] Liddle EB, Price D, Palaniyappan L, Brookes MJ, Robson SE, Hall EL, et al Abnormal salience signaling in schizophrenia: The role of integrative beta oscillations. Hum Brain Mapp. 2016;37(4):1361–74. 10.1002/hbm.23107 . 26853904 PMC4790909

[16] Palaniyappan L, Liddle PF. Does the salience network play a cardinal role in psychosis? An emerging hypothesis of insular dysfunction. J Psychiatry Neurosci. 2012;37(1):17–27. 10.1503/jpn.100176 . 21693094 PMC3244495

[17] den Ouden HE, Kok P, de Lange FP. How prediction errors shape perception, attention, and motivation. Front Psychol. 2012;3:548 10.3389/fpsyg.2012.00548 . 23248610 PMC3518876

[18] Leong YC, Radulescu A, Daniel R, DeWoskin V, Niv Y. Dynamic Interaction between Reinforcement Learning and Attention in Multidimensional Environments. Neuron. 2017;93(2):451–63. 10.1016/j.neuron.2016.12.040 . 28103483 PMC5287409

[19] Wunderlich K, Beierholm UR, Bossaerts P, O'Doherty JP. The human prefrontal cortex mediates integration of potential causes behind observed outcomes. Journal of neurophysiology. 2011;106(3):1558–69. 10.1152/jn.01051.2010 . 21697443 PMC3174823

[20] FitzGerald TH, Schwartenbeck P, Dolan RJ. Reward-related activity in ventral striatum is action contingent and modulated by behavioral relevance. The Journal of neuroscience: the official journal of the Society for Neuroscience. 2014;34(4):1271–9. 10.1523/JNEUROSCI.4389-13.2014 . 24453318 PMC3898287

[21] Dayan P, Hinton GE, Neal RM, Zemel RS. The helmholtz machine. Neural computation. 1995;7(5):889–904. 7584891 10.1162/neco.1995.7.5.889

[22] Friston K. A theory of cortical responses. Philos Trans R Soc Lond B Biol Sci. 2005;360(1456):815–36. 10.1098/rstb.2005.1622 . 15937014 PMC1569488

[23] Helmholtz H. Handbuch der physiologischen Optik. English translation (1962): Southall JPC, Dover, New York; 1860.

[24] Mathys C, Daunizeau J, Friston KJ, Stephan KE. A bayesian foundation for individual learning under uncertainty. Frontiers in human neuroscience. 2011;5:39 10.3389/fnhum.2011.00039 . 21629826 PMC3096853

[25] Adams RA, Huys QJ, Roiser JP. Computational Psychiatry: towards a mathematically informed understanding of mental illness. J Neurol Neurosurg Psychiatry. 2015 10.1136/jnnp-2015-310737 . 26157034 PMC4717449

[26] Adams RA, Stephan KE, Brown HR, Frith CD, Friston KJ. The computational anatomy of psychosis. Frontiers in psychiatry. 2013;4:47 10.3389/fpsyt.2013.00047 . 23750138 PMC3667557

[27] Fletcher PC, Frith CD. Perceiving is believing: a Bayesian approach to explaining the positive symptoms of schizophrenia. Nature reviews Neuroscience. 2009;10(1):48–58. 10.1038/nrn2536 . 19050712

[28] Heinz A, Schlagenhauf F. Dopaminergic Dysfunction in Schizophrenia: Salience Attribution Revisited. Schizophrenia bulletin 2010 p. 472–85. 10.1093/schbul/sbq031 20453041 PMC2879696

[29] Murray GK, Corlett PR, Clark L, Pessiglione M, Blackwell AD, Honey G, et al Substantia nigra/ventral tegmental reward prediction error disruption in psychosis. Molecular psychiatry. 2008;13(3):239, 67–76. 10.1038/sj.mp.4002058 . 17684497 PMC2564111

[30] Romaniuk L, Honey GD, King JR, Whalley HC, McIntosh AM, Levita L, et al Midbrain activation during Pavlovian conditioning and delusional symptoms in schizophrenia. Archives of general psychiatry. 2010;67(12):1246–54. 10.1001/archgenpsychiatry.2010.169 . 21135324

[31] Jensen J, Willeit M, Zipursky RB, Savina I, Smith AJ, Menon M, et al The formation of abnormal associations in schizophrenia: neural and behavioral evidence. Neuropsychopharmacology. 2008;33(3):473–9. 10.1038/sj.npp.1301437 . 17473838

[32] Holt DJ, Coombs G, Zeidan MA, Goff DC, Milad MR. Failure of neural responses to safety cues in schizophrenia. Archives of general psychiatry. 2012;69(9):893–903. 10.1001/archgenpsychiatry.2011.2310 . 22945619 PMC3767036

[33] Roiser JP, Stephan KE, den Ouden HE, Barnes TR, Friston KJ, Joyce EM. Do patients with schizophrenia exhibit aberrant salience? Psychological medicine. 2009;39(2):199–209. 10.1017/S0033291708003863 . 18588739 PMC2635536

[34] Roiser JP, Howes OD, Chaddock CA, Joyce EM, McGuire P. Neural and behavioral correlates of aberrant salience in individuals at risk for psychosis. Schizophrenia bulletin. 2013;39(6):1328–36. 10.1093/schbul/sbs147 . 23236077 PMC3796080

[35] Schmidt A, Antoniades M, Allen P, Egerton A, Chaddock CA, Borgwardt S, et al Longitudinal alterations in motivational salience processing in ultra-high-risk subjects for psychosis. Psychological medicine. 2017;47(2):243–54. 10.1017/S0033291716002439 . 27697078 PMC5216461

[36] Pankow A, Katthagen T, Diner S, Deserno L, Boehme R, Kathmann N, et al Aberrant Salience Is Related to Dysfunctional Self-Referential Processing in Psychosis. Schizophrenia bulletin. 2016;42(1):67–76. 10.1093/schbul/sbv098 . 26194892 PMC4681553

[37] Smieskova R, Roiser JP, Chaddock CA, Schmidt A, Harrisberger F, Bendfeldt K, et al Modulation of motivational salience processing during the early stages of psychosis. Schizophrenia research. 2015;166(1–3):17–23. 10.1016/j.schres.2015.04.036 . 25999039

[38] Abboud R, Roiser JP, Khalifeh H, Ali S, Harrison I, Killaspy HT, et al Are persistent delusions in schizophrenia associated with aberrant salience? Schizophr Res Cogn. 2016;4:32–8. 10.1016/j.scog.2016.04.002 . 27284531 PMC4884769

[39] Katthagen T, Dammering F, Kathmann N, Kaminski J, Walter H, Heinz A, et al Validating the construct of aberrant salience in schizophrenia—Behavioral evidence for an automatic process. Schizophrenia Research: Cognition. 2016;6:22–7. Epub 2016. 10.1016/j.scog.2016.10.001 28740821 PMC5514317

[40] Green MF, Harvey PD. Cognition in schizophrenia: Past, present, and future. Schizophr Res Cogn. 2014;1(1):e1–e9. 10.1016/j.scog.2014.02.001 . 25254156 PMC4171037

[41] Waltz JA, Gold JM. Motivational Deficits in Schizophrenia and the Representation of Expected Value. Curr Top Behav Neurosci. 2016;27:375–410. 10.1007/7854_2015_385 . 26370946 PMC4792780

[42] Bora E, Binnur Akdede B, Alptekin K. Neurocognitive impairment in deficit and non-deficit schizophrenia: a meta-analysis. Psychological medicine. 2017;47(14):2401–13. 10.1017/S0033291717000952 . 28468693

[43] Krkovic K, Moritz S, Lincoln TM. Neurocognitive deficits or stress overload: Why do individuals with schizophrenia show poor performance in neurocognitive tests? Schizophrenia research. 2017;183:151–6. 10.1016/j.schres.2016.11.002 . 27838097

[44] Diaconescu AO, Mathys C, Weber LA, Kasper L, Mauer J, Stephan KE. Hierarchical prediction errors in midbrain and septum during social learning. Social cognitive and affective neuroscience. 2017 10.1093/scan/nsw171 . 28119508 PMC5390746

[45] Mathys CD, Lomakina EI, Daunizeau J, Iglesias S, Brodersen KH, Friston KJ, et al Uncertainty in perception and the Hierarchical Gaussian Filter. Frontiers in human neuroscience. 2014;8:825 10.3389/fnhum.2014.00825 . 25477800 PMC4237059

[46] Rao RP, Ballard DH. Predictive coding in the visual cortex: a functional interpretation of some extra-classical receptive-field effects. Nat Neurosci. 1999;2(1):79–87. 10.1038/4580 . 10195184

[47] Stephan KE, Penny WD, Daunizeau J, Moran RJ, Friston KJ. Bayesian model selection for group studies. NeuroImage. 2009;46(4):1004–17. 10.1016/j.neuroimage.2009.03.025 . 19306932 PMC2703732

[48] Jessup RK, Busemeyer JR, Brown JW. Error effects in anterior cingulate cortex reverse when error likelihood is high. The Journal of neuroscience: the official journal of the Society for Neuroscience. 2010;30(9):3467–72. 10.1523/JNEUROSCI.4130-09.2010 . 20203206 PMC2841347

[49] Davidson MC, Horvitz JC, Tottenham N, Fossella JA, Watts R, Ulug AM, et al Differential cingulate and caudate activation following unexpected nonrewarding stimuli. NeuroImage. 2004;23(3):1039–45. 10.1016/j.neuroimage.2004.07.049 . 15528104

[50] Alexander WH, Brown JW. Medial prefrontal cortex as an action-outcome predictor. Nat Neurosci. 2011;14(10):1338–44. 10.1038/nn.2921 . 21926982 PMC3183374

[51] O'Reilly JX, Schuffelgen U, Cuell SF, Behrens TE, Mars RB, Rushworth MF. Dissociable effects of surprise and model update in parietal and anterior cingulate cortex. Proc Natl Acad Sci U S A. 2013;110(38):E3660–9. 10.1073/pnas.1305373110 . 23986499 PMC3780876

[52] Fernandez RS, Boccia MM, Pedreira ME. The fate of memory: Reconsolidation and the case of Prediction Error. Neuroscience and biobehavioral reviews. 2016;68:423–41. 10.1016/j.neubiorev.2016.06.004 . 27287939

[53] Guise KG, Shapiro ML. Medial Prefrontal Cortex Reduces Memory Interference by Modifying Hippocampal Encoding. Neuron. 2017;94(1):183–92 e8. 10.1016/j.neuron.2017.03.011 . 28343868 PMC5398284

[54] Lodge DJ, Grace AA. Hippocampal dysregulation of dopamine system function and the pathophysiology of schizophrenia. Trends Pharmacol Sci. 2011;32(9):507–13. 10.1016/j.tips.2011.05.001 . 21700346 PMC3159688

[55] Waltz JA. The neural underpinnings of cognitive flexibility and their disruption in psychotic illness. Neuroscience. 2016 10.1016/j.neuroscience.2016.06.005 . 27282085 PMC5143214

[56] Dowd EC, Frank MJ, Collins A, Gold JM, Barch DM. Probabilistic Reinforcement Learning in Patients With Schizophrenia: Relationships to Anhedonia and Avolition. Biol Psychiatry Cogn Neurosci Neuroimaging. 2016;1(5):460–73. 10.1016/j.bpsc.2016.05.005 . 27833939 PMC5098503

[57] Schlagenhauf F, Huys QJ, Deserno L, Rapp MA, Beck A, Heinze HJ, et al Striatal dysfunction during reversal learning in unmedicated schizophrenia patients. NeuroImage. 2014;89:171–80. 10.1016/j.neuroimage.2013.11.034 . 24291614 PMC3991847

[58] Culbreth AJ, Westbrook A, Xu Z, Barch DM, Waltz JA. Intact Ventral Striatal Prediction Error Signaling in Medicated Schizophrenia Patients. Biol Psychiatry Cogn Neurosci Neuroimaging. 2016;1(5):474–83. 10.1016/j.bpsc.2016.07.007 . 28239676 PMC5321567

[59] Ballard I, Miller EM, Piantadosi ST, Goodman ND, McClure SM. Beyond Reward Prediction Errors: Human Striatum Updates Rule Values During Learning. Cereb Cortex. 2017:1–11. Epub 2017/10/19. 10.1093/cercor/bhx259 . 29040494 PMC6685076

[60] Holt DJ, Titone D, Long LS, Goff DC, Cather C, Rauch SL, et al The misattribution of salience in delusional patients with schizophrenia. Schizophrenia research. 2006;83(2–3):247–56. 10.1016/j.schres.2005.12.858 . 16540291

[61] Radua J, Schmidt A, Borgwardt S, Heinz A, Schlagenhauf F, McGuire P, et al Ventral Striatal Activation During Reward Processing in Psychosis: A Neurofunctional Meta-Analysis. JAMA Psychiatry. 2015;72(12):1243–51. 10.1001/jamapsychiatry.2015.2196 . 26558708

[62] Diaconescu AO, Jensen J, Wang H, Willeit M, Menon M, Kapur S, et al Aberrant Effective Connectivity in Schizophrenia Patients during Appetitive Conditioning. Frontiers in human neuroscience. 2011;4:239 10.3389/fnhum.2010.00239 . 21267430 PMC3024844

[63] Schmidt K, Roiser JP. Assessing the construct validity of aberrant salience. Frontiers in behavioral neuroscience. 2009;3:58 10.3389/neuro.08.058.2009 . 20057930 PMC2802547

[64] Daberkow DP, Brown HD, Bunner KD, Kraniotis SA, Doellman MA, Ragozzino ME, et al Amphetamine paradoxically augments exocytotic dopamine release and phasic dopamine signals. The Journal of neuroscience: the official journal of the Society for Neuroscience. 2013;33(2):452–63. 10.1523/JNEUROSCI.2136-12.2013 . 23303926 PMC3711765

[65] Boehme R, Deserno L, Gleich T, Katthagen T, Pankow A, Behr J, et al Aberrant Salience Is Related to Reduced Reinforcement Learning Signals and Elevated Dopamine Synthesis Capacity in Healthy Adults. The Journal of neuroscience: the official journal of the Society for Neuroscience. 2015;35(28):10103–11. 10.1523/JNEUROSCI.0805-15.2015 . 26180188 PMC6605337

[66] Marshall L, Mathys C, Ruge D, de Berker AO, Dayan P, Stephan KE, et al Pharmacological Fingerprints of Contextual Uncertainty. PLoS Biol. 2016;14(11):e1002575 10.1371/journal.pbio.1002575 . 27846219 PMC5113004

[67] Vossel S, Mathys C, Daunizeau J, Bauer M, Driver J, Friston KJ, et al Spatial attention, precision, and Bayesian inference: a study of saccadic response speed. Cereb Cortex. 2014;24(6):1436–50. 10.1093/cercor/bhs418 . 23322402 PMC4014178

[68] Lawson RP, Mathys C, Rees G. Adults with autism overestimate the volatility of the sensory environment. Nat Neurosci. 2017;20(9):1293–9. 10.1038/nn.4615 . 28758996 PMC5578436

[69] Vossel S, Mathys C, Stephan KE, Friston KJ. Cortical Coupling Reflects Bayesian Belief Updating in the Deployment of Spatial Attention. The Journal of neuroscience: the official journal of the Society for Neuroscience. 2015;35(33):11532–42. 10.1523/JNEUROSCI.1382-15.2015 . 26290231 PMC4540794

[70] Silverstein SM, Keane BP. Perceptual organization impairment in schizophrenia and associated brain mechanisms: review of research from 2005 to 2010. Schizophrenia bulletin. 2011;37(4):690–9. 10.1093/schbul/sbr052 . 21700589 PMC3122298

