# Supplementary material for: Modeling subjective relevance in schizophrenia and its relation to aberrant salience
Source: PLoS Comput Biol. 2018 Aug 10;14(8):e1006319. doi: 10.1371/journal.pcbi.1006319 (PMC6105009; doi:10.1371/journal.pcbi.1006319)
Supplement: S1 Text — The supplement contains the following analyses and results: additional information on the ISP and raw data analyses of potential stimulus feature effects, additional information on the HGF and the HGF-precision feedback learning model, overview of the model space, fMRI preprocessing, and tables of the priors of learning and response models. (DOCX) [file pcbi.1006319.s001.docx]

**Supplemental Information**

**Modeling subjective relevance in schizophrenia and its relation to aberrant salience**

**Teresa Katthagen, Christoph Mathys, Lorenz Deserno, Henrik Walter, Norbert Kathmann, Andreas Heinz, Florian Schlagenhauf**

1. **Additional information on the ISP and raw data analyses**

**Performance on the ISP.** In the ISP, two reward contingencies of the two features, shape and color, can be learned, whereas only one feature reliably predicts reward. Performance in terms of correct responses to the outcome was high in both groups (median percent correct: HC=99.4, Sz=98.8, *Mann-Whitney-U*=625.5, p=.018) as well as the median response rate in general (HC=100, Sz =100).

**Addressing potential influences of cue feature on learning.** In these analyses, we investigated whether participants responded differently towards certain cue features (color, gray, triangle and square) and whether the different cue features interacted with the learning process. First, we tested if participants were overall faster for certain cue features using a repeated-measures ANOVA with cue feature as within and group as between subject factor. Individual mean reaction times showed no significant main effect of cue feature (p>0.29) and no significant interaction with group (p>0.84). To test if cue features interacted with the observed learning effects we introduced cue feature as an additional within-subject factor into the repeated measures ANOVAs as described in the main manuscript (Section 3: Raw data analysis). The event type* cue feature*group ANOVA showed no significant main effect of cue feature (*F*=0.3, *p*>0.8) nor a significant interaction between feature and event type (*F*=1.4, *p*>.2) or feature*group (*F*=0.02, *p*>0.8), while event type remained significant (*F*=5.9, *p*=0.018). In a separate time*reward*group*cue feature ANOVA we tested if participants speeded up their responses after a contingency change. The cue feature factor was introduced into this ANOVA by grouping the reaction time bins into 4 blocks according to the respectively relevant cue feature for predicting rewards and circles. In line with our ANOVA reported in the manuscript, subjects speeded up their responses over time bins (two bins spanning 4 consecutive trials, each for coin and circle) within one run of 16 non-probabilistic trials (main effect time: *F*=6.08, *p*=.016) and were faster for coins than for circles (main effect reward: F=22.767, p<0.001). There were no significant effects for the relevant cue feature (main effect cue feature: *p*>0.4; interaction cue feature * group: *p*>0.9; interaction cue feature * time: *p*>.11; cue feature * time * reward: *p* > 0.24).

**Test half analyses.** We followed one reviewer’s suggestion and investigated whether the extra-dimensional shift in the middle of the experiment affected subjects’ behavior. Following the first 80 trials of the experiment, the previously relevant dimension turned irrelevant and vice versa. We tested for potential effects of this shift by adding ‘Test half’ as a within-subject factor into the Reward*Time*Group ANOVA of the log-reaction times (please compare with the raw data analysis in the Manuscript, p. 9-10). There was no significant main effect of Test half (F=.197, p=.687) nor a significant interaction of Test half with time and/or group (F=.588, p=.623; F=.805 for Test half*Time; p=.492 for Test half*Time*Group) indicating that the switch did not affect subjects’ learning. We assume that this might have been different if the switch had appeared more often and/or if it had been explicitly instructed, as in intra-dimension/extra-dimension paradigms [for a review see 1].

1. **Antipsychotic medication status and control analyses**

All patients of the current study received antipsychotic medication. Detailed information on substance, dosage and medication history of the individual patients are displayed in **Table A**.

**Table A. Medication in schizophrenia patients.**

| **Patient** | **Medication (dose)** | **Since when?** |
| --- | --- | --- |
| *No. 1* | Amisulpride (40 mg) | 1 year |
| *No. 2* | Risperdone (7 mg); Quetiapine (25 mg) | No information |
| *No. 3* | Olenzapine (7.5 mg) | No information |
| *No. 4* | Ziprasidone (60 mg), Promethazine (50 mg), Queliapine (100 mg) | No information |
| *No. 5* | Olanzapine (20 mg) | 10 years |
| *No. 6* | Olanzapine (15 mg), Aripiprazole (10 mg) | 6 years |
| *No. 7* | Aripiprazole (15 mg), Risperdone (0.5 mg) | 1 month |
| *No. 8* | Quetiapine (600 mg) | No information |
| *No. 9* | Amisulpride (200 mg); Quetiapine (200 mg) | 13 months; 9 months |
| *No. 10* | Olanzapine (20 mg) | No information |
| *No. 11* | Amisulpride (300 mg), Quetiapine (200 mg) | 8 months; 7 years |
| *No. 12* | Amisulpride (400 mg) | No information |
| *No. 14* | Risperdone (3 mg) | 2 weeks |
| *No. 15* | Aripiprazole (2.5 mg) | 5 years |
| *No. 16* | Risperdone (4.5 mg) | 1 month |
| *No. 17* | Risperdone (4 mg) | 3 years |
| *No. 18* | Olanzapine (10 mg) | 1 month |
| *No. 19* | Clozapine (125 mg), Amisulpride (200-300 mg) | No information |
| *No. 20* | Amisulpride (400 mg), Olanzapine (5 mg) | 1 year |
| *No. 22* | Quetiapin (200 mg), Amisulprid (1000 mg) | No information |
| *No. 23* | Ziprasidone (120 mg) | 6 years |
| *No. 24* | Amisulpride (100 mg), Citalopram (30 mg) | 1.5 years |
| *No. 25* | Amisulpride (100 mg) | 4 months |
| *No. 26* | Risperdone (1.25 mg) | 5 years |
| *No. 27* | Clozapine (150 mg) | 1.5 months |
| *No. 28* | Paliperidone (100 mg) | 3 months |
| *No. 29* | Flunoaxol (60 mg) | 2-3 years |
| *No. 30* | Risperdone (4 mg) | 7 months |
| *No. 31* | Aripiprazole (10 mg) | 9 years |
| *No. 32* | Clozapine (300 mg) | 13 years |
| *No. 33* | Amisulpride (150 mg), Clozapine (125 mg) | 16 years |
| *No. 34* | Aripiprazole (15 mg) | 1 year |
| *No. 35* | Clozapine (250 mg) | 7 years |
| *No. 36* | Haloperidol | 3-4 months |
| *No. 37* | Paliperidone (50 mg) | 13 months |
| *No. 38* | Aripiprazole (20 mg) | 3 weeks |
| *No. 39* | Risperdone (6 mg) | 4 weeks |
| *No. 40* | Paliperidone (150 mg) | 3 months |
| *No. 41* | Risperdone (4 mg) | No information |
| *No. 42* | Risperdone (4 mg) | No information |

In order to test for a potentially confounding effect of antipsychotic medication on aberrant salience attribution, we correlated chlorpromazine equivalent scores with (1) aberrant salience (ground truth) and (2) β_irrelevance. There were no significant correlations between ISP scores and CPZ equivalents (*p*>0.8). Further, including chlorpromazine equivalent scores as a covariate to control for medication effects, the positive correlation between nucleus accumbens response and beta_irrelevance remained significant ([-14 6 -10], *t*=4.83, *p*_SVCB_=0.003).

1. **Control analyses for IQ**

Patients and healthy individuals differed in their verbal IQ. Thus, we performed additional group comparisons on the ground truth aberrant salience scores as well as on the beta_irrelevance measures while controlling for verbal IQ. There was no confounding effect by IQ, since groups still differed significantly on both measures (ground truth aberrant salience: *F*=3.75, *p*=0.029; β_irrelevance values: *F*=6.26, *p*=0.015) in these analyses.

1. **Additional information on the learning models: HGF and HGF-precision feedback**

**Hierarchical Gaussian Filter.** In our model space, we varied the maximum of levels between 2 and 3. On the first level, the association of stimulus with outcome is coded. For example, when at the $k$th trial a colorful stimulus is followed by a reward, this is coded as $x_{1}^{k}=1$. When a colorful stimulus is followed by a neutral outcome, this is coded as $x_{1}^{k}=0$. Since for gray stimuli the probabilistic association with outcome is reversed, the coding is, too. That is to say, when a gray stimulus is followed by a reward, this is coded as $x_{1}^{k}=0$, and when it is followed by a neutral outcome as $x_{1}^{k}=1$. The same kind of coding as in the color domain applies to the shape domain. Since associations are probabilistic and therefore bounded between 0 and 1, the probability of $x_{1}^{k}$ being 1 or 0 is modeled as the sigmoidal transformation of a second-level tendency$x_{2}^{k}$ as in Iglesias et al. [2].

1. $p\left( x_{1} | x_{2} \right)=s{(x_{2})}^{x1}(1-s{{(x}_{2}))}^{1-x1}$

On the second level, predictions of stimulus-outcome associations are learned as in the ISP where the relevant stimulus features predicted reward or neutral outcomes in a probabilistic manner. Second-level learning is defined as a Gaussian random walk around $x_{2}$ depending on the previous estimate of $x_{2}^{(k-1)}$ and the current estimate of the third level $x_{3}^{(k)}$(see 4). In our model space, differences in learning can be captured by the free parameters *ω* that determines the constant of the step size (being similar to a constant learning rate) and the initial uncertainty$\sigma_{2}^{0}$. To reduce the complexity of the model, these parameters were the same for color and shape learning. The parameter *κ* that determines the coupling strength was fixed to 1 for a full 3-level HGF (**3HGF**) and to 0 for a 2-level HGF (**2HGF**) where the influence of the third level was eliminated.

$$p\left( x_{2}^{(k)} | x_{2}^{(k-1)},x_{3}^{k} \right)\mathcal{=N}\left( x_{2}^{(k)};x_{2}^{\left( k-1 \right)},exp({\kappa x}_{3}^{\left( k \right)}+\omega) \right)$$

The third level predictions track the volatility of the environment or, in the context of our paradigm, the subjective estimation of second-level predictions’ stability. Since there is no higher level in our model space, its update is equally determined as on the previous level, but the Gaussian walk’s step size is only determined by the parameter $\vartheta$ that was set to exp(-6)=0.0025 here. In all **3HGF** models, there was an autoregressive tendency $\varphi_{3}$back to the starting point of $\mu_{3}^{(0)}$preventing large drifts in third level beliefs. This prevented ceiling effects in the third level belief trajectories and thus led to more plausible trajectories in light of the fact that there were objectively no changes in the volatility of contingency.

$$p\left( x_{3}^{k} | x_{3}^{k-1},\vartheta\right)\mathcal{=N}\left( x_{3}^{(k)}; x_{3}^{(k-1)}+ \varphi_{3}\left( \mu_{3}^{(0)}-x_{3}^{(k-1)} \right),\vartheta\right)$$

**Precision Feedback**

Here, we describe how we implemented the idea that relevance affects learning by letting the relevance/first level precision $\hat{\pi}_{1}^{\left( k \right)}$ affect the second level HGF update. First-level precisions may be used to regulate trial-by-trial updates on the second level, leading to decreased update in the uninformative prediction and increased in the informative prediction. The influence of this ‘precision feedback’ (precfb) is expressed by the free parameter$\varphi_{1}$.

1. $p\left( x_{2}^{(k)} | x_{2}^{(k-1)},x_{3}^{k} \right)\mathcal{=N}\left( x_{2}^{(k)};x_{2}^{\left( k-1 \right)},\exp\left( {\kappa x}_{3}^{\left( k \right)}+\omega\right)+ \varphi_{1}(\hat{\pi}_{1}^{\left( k \right)}-4) \right)$

**
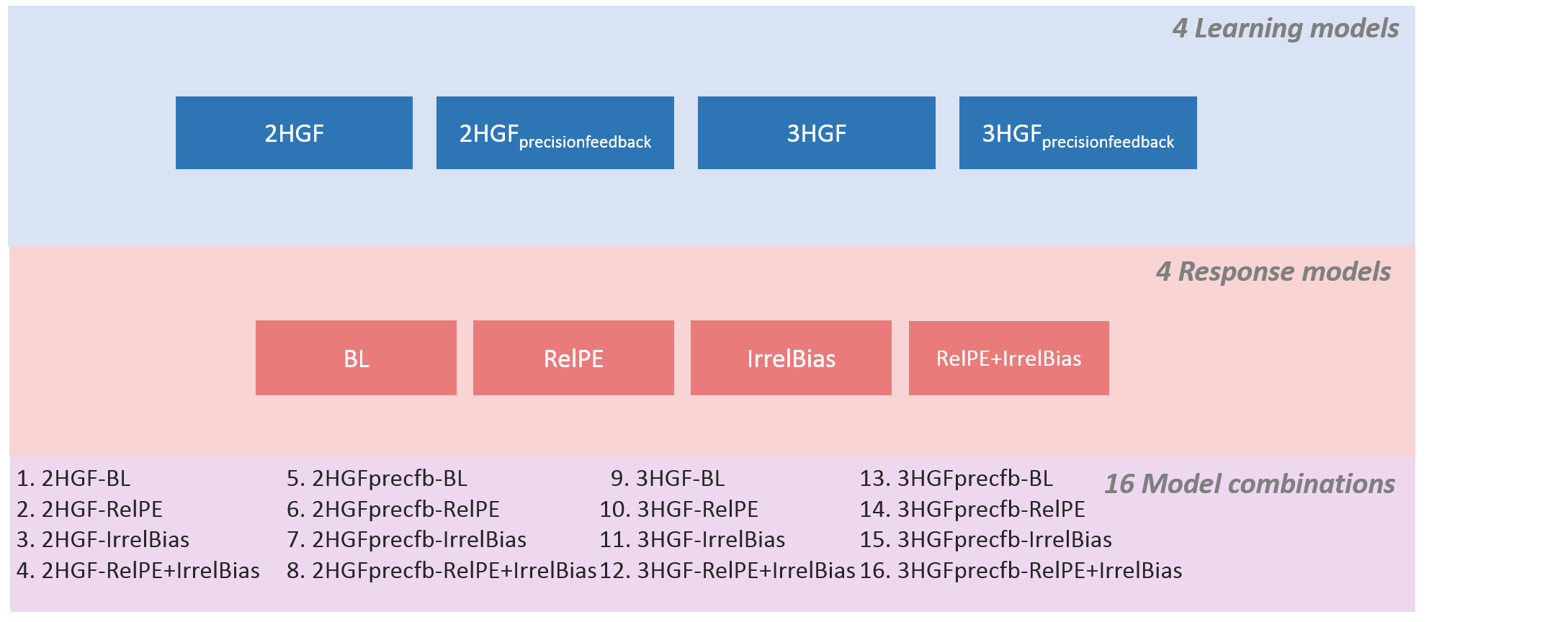
**

**Figure A. Model Space.**

**Table B. Parameter priors of the HGF variants and response models.** Priors with a variance=0 were fixed.

| **Prior Mean (variance) of** | **2HGF** | **2HGFprecfb** | **3HGF** | **3HGFprecfb** |
| --- | --- | --- | --- | --- |
| $\mu_{2}^{0}; \mu_{3}^{0}$ | 0(0); 0(0) | 0(0); 0(0) | 0(0); 0(0) | 0(0); 0(0) |
| $\sigma_{2}^{0}; \sigma_{3}^{0}$ (in log-space) | 0.05 (1); 0 (0) | 0.05 (1); 0 (0) | 0.05 (1); 0 (0) | 0.05 (1); 0 (0) |
| $\varphi_{1}, \varphi_{2}$ (in logit-space) | -;- | .06 (1) ; - | -; 0.1 (0) | .06(1)/0.1 (0) |
| $\kappa$ (in log-space) | 0 (0) | 0 (0) | 1 (0) | 1 (0) |
| $\omega$ | -2 (.25) | -2 (.25) | -2 (.25) | -2 (.25) |
| $\vartheta$ | log(-6) (0) | log(-6) (0) | log(-6) (0) | log(-6) (0) |
|  |  | | | |
|  | **Response models (all parameters estimated in log-space)** | | | |
| $\beta_{0}$ | 500 (1) | | | |
| $\beta_{1}$ | 1 (4) | | | |
| $\beta_{2}$ | 0.1 (1) | | | |
| $\beta_{3}$ | 0.1 (1) | | | |
| $\beta_{4}$ | 0.1 (1) | | | |
| $\beta_{5}$ | 0.1 (1) | | | |
| $\beta_{6}$ | 0.05 (1) | | | |
| $\zeta$ | log(3) (log(2)) | | | |

**Table C.** Model fit criteria (AIC=Akaide Information criterion and BIC=Bayesian Information criterion) of the 16 model combinations.

|  |  | **2HGF-BL** | **2HGF-relPE** | **2HGF-irrelBias** | **2HGF-full** | **2HGFprecfb-BL** | **2HGFprecfb-relPE** | **2HGFprecfb-irrelBias** | **2HGFprecfb-full** | **3HGF-BL** | **3HGF-relPE** | **3HGF-irrelBias** | **3HGF-full** | **3HGFprecfb-BL** | **3HGFprecfb-relPE** | **3HGFprecfb-irrelBias** | **3HGFprecfb-full** |
| --- | --- | --- | --- | --- | --- | --- | --- | --- | --- | --- | --- | --- | --- | --- | --- | --- | --- |
| **Full sample** | **AIC** | -82.6 | -82.7 | -83.6 | **-83.7** | -80.9 | -81.1 | -82.2 | -82.3 | -82.6 | -82.7 | -83.6 | -83.7 | -80.6 | -81.1 | -82.1 | -82.3 |
|  | **BIC** | -51.9 | -52.1 | -52.9 | **-53** | -47.2 | -47.3 | -48.4 | -48.5 | -51.9 | -52.1 | -52.9 | -53 | -46.9 | -47.3 | -48.4 | -48.5 |
| **Only HC** | **AIC** | -107 | -107.1 | -108 | **-108.1** | -105.3 | -105.4 | -106.4 | -106.7 | -107 | -107.1 | -108 | -108.1 | -105 | -105.5 | -106.5 | -106.7 |
|  | **BIC** | -76.3 | -76.4 | -77.3 | **-77.4** | -71.5 | -71.7 | -72.6 | -72.9 | -76.3 | -76.4 | -77.3 | -77.4 | -71.2 | -71.7 | -72.7 | -72.9 |
| **Only Sz** | **AIC** | -58.3 | -58.4 | -59.1 | **-59.2** | -56.5 | -56.7 | -57.9 | -57.9 | -58.3 | -58.4 | -59.1 | -59.2 | -56.3 | -56.7 | -57.7 | -57.9 |
|  | **BIC** | -27.6 | -27.7 | -28.5 | **-28.6** | -22.8 | -23 | -24.2 | -24.2 | -27.6 | -27.7 | -28.5 | -28.6 | -22.6 | -23 | -24 | -24.2 |

HC=healthy controls; Sz=schizophrenia patients; BL=Baseline model; relPE=relevance weighted prediction error; irrelBias = irrelevance weighted bias; full = full response model incl. relevance weighted PE and irrelevance bias;

1. **Preprocessing of fMRI data**

We performed functional magnetic resonance imaging using a 3 Tesla Siemens Trio scanner to acquire gradient echo T2*-weighted echo-planar images with blood oxygenation level dependent contrast. Covering the whole brain, 36 slices were acquired in oblique orientation at 25° to AC-PC line in interleaved order with 2.5-mm thickness and 3x3mm² in-plane voxel resolution. The gap between slices was 0.5-mm, the echo time had a TR=2s and a TE=22ms and a flip angle α=90°. In addition, T1-weighted structural images were acquired (TR=1300ms, TE=3.46ms, flip=10°, matrix=240×256, voxel size: 1×1×1mm, slices=170) as well as a field map prior to functional scanning to account for individual homogeneity differences of the magnetic field. After preprocessing, due to increased movement during scanning (> 3 mm on x, y or z axis) 4 subjects (2 HC and 2 patients) were excluded from further analysis. Four further subjects excluded from analysis due to scanning artifacts (2 HC and 2 patients), leading to 38 healthy individuals and 38 schizophrenia patients in our final sample for fMRI analysis.

1. **Correlations and potential transfer effects analysis regarding the Salience Attribution Test [3]**

Of the current study, 39 healthy controls and 39 schizophrenia patients also performed the Salience Attribution Test in an additional behavioral session. Most of these data (37 HC and 34 Sz) were part of a construct validity analysis reported in our previous manuscript [4]. In **Table C** we report the measures of the SAT and include ISP measures as reported in the manuscript. Correlations between those measures are reported in **Table D** (patients) and **E** (controls).

In the SAT and compared to healthy controls, schizophrenia patients showed decreased scores for implicit (reaction time based) and explicit (judgement on a visual-analogue scale based) adaptive salience, as well as increased implicit aberrant salience.

We tested for correlations between salience measures from the SAT and modeling parameters and the aberrant salience raw data score from the ISP (see **Table D and E**). In healthy individuals, there were no significant correlations between SAT and ISP scores (for within task correlations, please see **Table C**). In schizophrenia patients, the free modeling parameter beta_irrelevance correlated with implicit aberrant salience from the SAT and there was a trendwise positive correlation between the latter and ground truth aberrant salience from the ISP.

**Table D. Salience attribution from the SAT and ISP.**

|  | **Healthy controls** | **Schizophrenia patients** | **t-statistic** | **p-value** |
| --- | --- | --- | --- | --- |
| **SAT Adaptive Salience (implicit)** | 17.99 ± 14.57 | 9.00 ± 20.36 | 2.21 | .030 |
| **SAT Adaptive Salience (explicit)** | 58.11 ± 21.72 | 33.44 ±27.33 | 4.36 | < 0.001 |
| **SAT Aberrant Salience (implicit)** | 3.27 ±1.06 | 3.99 ± 1.47 | -2.49 | 0.015 |
| **SAT Aberrant Salience (explicit)** | 2.24 ± 1.50 | 2.66 ± 1.14 | -1.38 | 0.173 |
| **ISP Aberrant Salience** | 17.98 ± 10.88 | 22.95 ± 14.46 | -1.78 | 0.0395 |
| **ISP β _irrelevance** | 0.037 ± 0.019 | 0.0499 ± 0.027 | -2.56 | 0.012 |
| **ISP β _1** | 0.204 ± 0.010 | 0.212 ± 0,101 | -.38 | 0.706 |

Since subjects were explicitly instructed in the SAT to judge the amount of reward for each cue type on a visual analogue scale, subjects might have transferred this knowledge of inferences between cues and feedback to the ISP, where such contingencies were not instructed. However, there was no evidence of potential transfer or generalization effects in the data. First, only 19 participants (9 HC, 10 Sz, 3 HC and 3 Sz had no SAT session) performed the SAT in our behavioral and neuropsychology session *before* they underwent fMRI with the ISP (median days in between: 4 ± 7.7 days). Second, aberrant salience ISP scores (based on the ground truth) and β_irrelevance values did not differ when we comparing subjects who performed the SAT before the ISP with those who performed it the other way around (((1) total sample: aberrant salience (ground truth), t(82)=.8, p=.42; β_irrelevance, t(82)=1.2, p=.229; (2) only HC: aberrant salience (ground truth), t(40)=.43, p=.672; β_irrelevance, t(40)=.81, p=.423; (3) only Sz: aberrant salience (ground truth), t(40)=.76, p=.543; β_irrelevance, t(40)=1.04, p=.306)). Lastly, when we run the reaction times-ANOVA from the main manuscript in patients while including the between subjects factor “SAT before ISP” (0 vs. 1), there was no evidence for better learning in patients who performed the SAT before the ISP (time*”SAT before ISP” interaction *p* > .11).

**Table E. Correlations between task measures from the SAT and ISP in healthy individuals.**

|  |  | **1. SAT Adaptive Salience (implicit)** | **2. SAT Adaptive Salience (explicit)** | **3. SAT Aberrant Salience (implicit)** | **4. SAT Aberrant Salience (explicit)** | **5. ISP Aberrant Salience** | **6. ISP β_irrelevance** | **7. ISP** **β_1** |
| --- | --- | --- | --- | --- | --- | --- | --- | --- |
| **1.** | *Spearman’s rho* | 1.000 | 0.248 | **.413** | -0.021 | -0.240 | -0.115 | -0.306 |
|  | *p* |  | 0.139 | **0.011** | 0.903 | 0.152 | 0.499 | 0.066 |
| **2.** |  |  | 1.000 | 0.152 | **-0.578** | -0.285 | -0.199 | -0.121 |
|  |  |  |  | 0.368 | **<0.001** | 0.087 | 0.237 | 0.475 |
| **3.** |  |  |  | 1.000 | -0.278 | 0.134* | 0.247* | 0.136 |
|  |  |  |  |  | 0.095 | 0.215 | 0.071 | 0.422 |
| **4.** |  |  |  |  | 1.000 | 0.135 | 0.042 | -0.139 |
|  |  |  |  |  |  | 0.425 | 0.620 | 0.412 |
| **5.** |  |  |  |  |  | 1.000 | **0.523** | 0.073 |
|  |  |  |  |  |  |  | **<0.001** | 0.645 |
| **6.** |  |  |  |  |  |  | 1.000 | 0.265 |
|  |  |  |  |  |  |  |  | 0.090 |
| **7.** |  |  |  |  |  |  |  | 1.000 |
|  |  |  |  |  |  |  |  |  |

*tested one-tailed based on our a priori hypothesis of positive correlations between implicit aberrant salience measures between tasks.

**Table F. Correlations between task measures from the SAT and ISP in schizophrenia patients.**

|  |  | **1. SAT Adaptive Salience (implicit)** | **2. SAT Adaptive Salience (explicit)** | **3. SAT Aberrant Salience (implicit)** | **4. SAT Aberrant Salience (explicit)** | **5. ISP Aberrant Salience** | **6. ISP β_irrelevance** | **7. ISP β_1** |
| --- | --- | --- | --- | --- | --- | --- | --- | --- |
| **1.** | *Spearman’s rho* | 1.000 | 0.262 | **.345** | -0.048 | 0.036 | 0.260 | -0.050 |
|  | *p* |  | 0.103 | **0.029** | 0.767 | 0.825 | 0.106 | 0.759 |
| **2.** |  |  | 1.000 | -0.094 | -0.016 | -0.051 | 0.063 | 0.066 |
|  |  |  |  | 0.563 | 0.922 | 0.756 | 0.698 | 0.685 |
| **3.** |  |  |  | 1.000 | -0.165 | **0.229*** | **0.285*** | -0.081 |
|  |  |  |  |  | 0.309 | **0.078** | **0.038** | 0.619 |
| **4.** |  |  |  |  | 1.000 | -0.066 | -0.213 | 0.047 |
|  |  |  |  |  |  | 0.686 | 0.186 | 0.774 |
| **5.** |  |  |  |  |  | 1.000 | **.612** | -0.075 |
|  |  |  |  |  |  |  | **<0.001** | 0.638 |
| **6.** |  |  |  |  |  |  | 1.000 | -0.010 |
|  |  |  |  |  |  |  |  | 0.952 |
| **7.** |  |  |  |  |  |  |  | 1.000 |
|  |  |  |  |  |  |  |  |  |

*tested one-tailed based on our a priori hypothesis of positive correlations between implicit aberrant salience measures between tasks.

References

1. Waltz JA. The neural underpinnings of cognitive flexibility and their disruption in psychotic illness. Neuroscience. 2016. doi: 10.1016/j.neuroscience.2016.06.005. PubMed PMID: 27282085.

2. Iglesias S, Mathys C, Brodersen KH, Kasper L, Piccirelli M, den Ouden HE, et al. Hierarchical prediction errors in midbrain and basal forebrain during sensory learning. Neuron. 2013;80(2):519-30. doi: 10.1016/j.neuron.2013.09.009. PubMed PMID: 24139048.

3. Roiser JP, Stephan KE, den Ouden HE, Barnes TR, Friston KJ, Joyce EM. Do patients with schizophrenia exhibit aberrant salience? Psychological medicine. 2009;39(2):199-209. doi: 10.1017/S0033291708003863. PubMed PMID: 18588739; PubMed Central PMCID: PMC2635536.

4. Katthagen T, Dammering F, Kathmann N, Kaminski J, Walter H, Heinz A, et al. Validating the construct of aberrant salience in schizophrenia - Behavioral evidence for an automatic process. Schizophrenia Research: Cognition. 2016;6:22-7. Epub 2016. doi: 10.1016/j.scog.2016.10.001.
